# Gaze distribution analysis and saliency prediction across age groups

**DOI:** 10.1371/journal.pone.0193149

**Published:** 2018-02-23

**Authors:** Onkar Krishna, Andrea Helo, Pia Rämä, Kiyoharu Aizawa

**Affiliations:** 1 Dept. of Information and Communication Engineering, The University of Tokyo, Tokyo, Japan; 2 Laboratoire Psychologie de la Perception, Université Paris Descartes, Paris, France; 3 Departamento de Fonoaudiología, Universidad de Chile, Santiago, Chile; 4 CNRS (UMR 8242), Paris, France; Huazhong University of Science and Technology, CHINA

## Abstract

Knowledge of the human visual system helps to develop better computational models of visual attention. State-of-the-art models have been developed to mimic the visual attention system of young adults that, however, largely ignore the variations that occur with age. In this paper, we investigated how visual scene processing changes with age and we propose an age-adapted framework that helps to develop a computational model that can predict saliency across different age groups. Our analysis uncovers how the explorativeness of an observer varies with age, how well saliency maps of an age group agree with fixation points of observers from the same or different age groups, and how age influences the center bias tendency. We analyzed the eye movement behavior of 82 observers belonging to four age groups while they explored visual scenes. Explorative- ness was quantified in terms of the entropy of a saliency map, and area under the curve (AUC) metrics was used to quantify the agreement analysis and the center bias tendency. Analysis results were used to develop age adapted saliency models. Our results suggest that the proposed age-adapted saliency model outperforms existing saliency models in predicting the regions of interest across age groups.

## Introduction

Computational models of human visual attention are becoming increasingly important, and investigations of these have driven much research by psychologists, neurobiologists and researchers in computer vision. The problem of predicting a region of a scene that attracts the observer remains a core challenge in vision research, that can at present be solved in two ways: using eye-tracking devices, like the TobiiX50 and Eyelink1000 and, by developing a computational model [1], [2], [3], [4] to mimic human vision for scene-viewing. Although eye trackers achieve high prediction accuracy, they are not always an in-hand option [4]. Thus, the use of computational models has gained an importance in the last few decades.

The era of the development of computational models was heralded by the pioneering work of Itti et al. [3] based on Treisman’s feature integration theory (FIT) [5], where a master saliency map is obtained by combining bottom-up feature maps in parallel. A series of works [6], [7], [8], [9], [10], [11] have since investigated similar issues, where the major differences laid in the way the features were selected and maps were combined. Some models integrated the maps linearly whereas others used non-linear techniques to combine them [9], [10]. A next set of computational models [12], [13] combined bottom-up features with top-down factors, such as, the given task [14], human tendency [15], habituation and conditioning [16], and emotions [17] as these factors are closely related to visual attention during scene viewing.

In recent years, vision research studies have investigated the role of human’s physical factors such as age, visual disparity, eye-sight, and gender, in driving human’s attention during scene viewing. Developmental studies suggest that age-related changes in eye movement control such as capability to fixate at target improves extensively during early childhood [18], [19], [20], [21] but more complex aspects of the fixation system, such as steadiness of fixations and cognitive control continues to develop until adolescence [19]. It has been also found that the saccades are shorter and less precise in children than adults [19] and that cognitive control of saccade execution, reaches an adult-like performance level at around 10–12 years of age [22], [23], [24].

Supporting evidence from developmental studies [25], [26] on scene exploration has also shown that there are remarkable differences in the scene-viewing behavior of observers across different age groups. For example, local image features, such as color, intensity, luminance, etc., were shown to guide fixation landings early in life, while later, fixation landings are dominated by more top-down processing [25], [26].

In spite of a few studies reporting developmental changes in scene viewing behavior, there are no studies that have systemically analyzed the gaze landing of observers across age groups in context of developing an age-adapted computational models. So far, the computational models have relied on the gaze data collected from adult participants but due to significant changes in visual skills during the development, it is essential to include also age factor in computational models.

Thus, computational models that have been developed until now compromise on prediction accuracy when different age groups are considered. Our study aims to parameterize the scene viewing tendency across age groups and to develop a new computational model that includes observer’s age in predicting salient locations for images. Our work is strategically beneficial, as most conventional models of visual attention can be easily tuned to age-related changes in scene viewing by following the recommendations of the results of our analysis.

**Fig 1:**
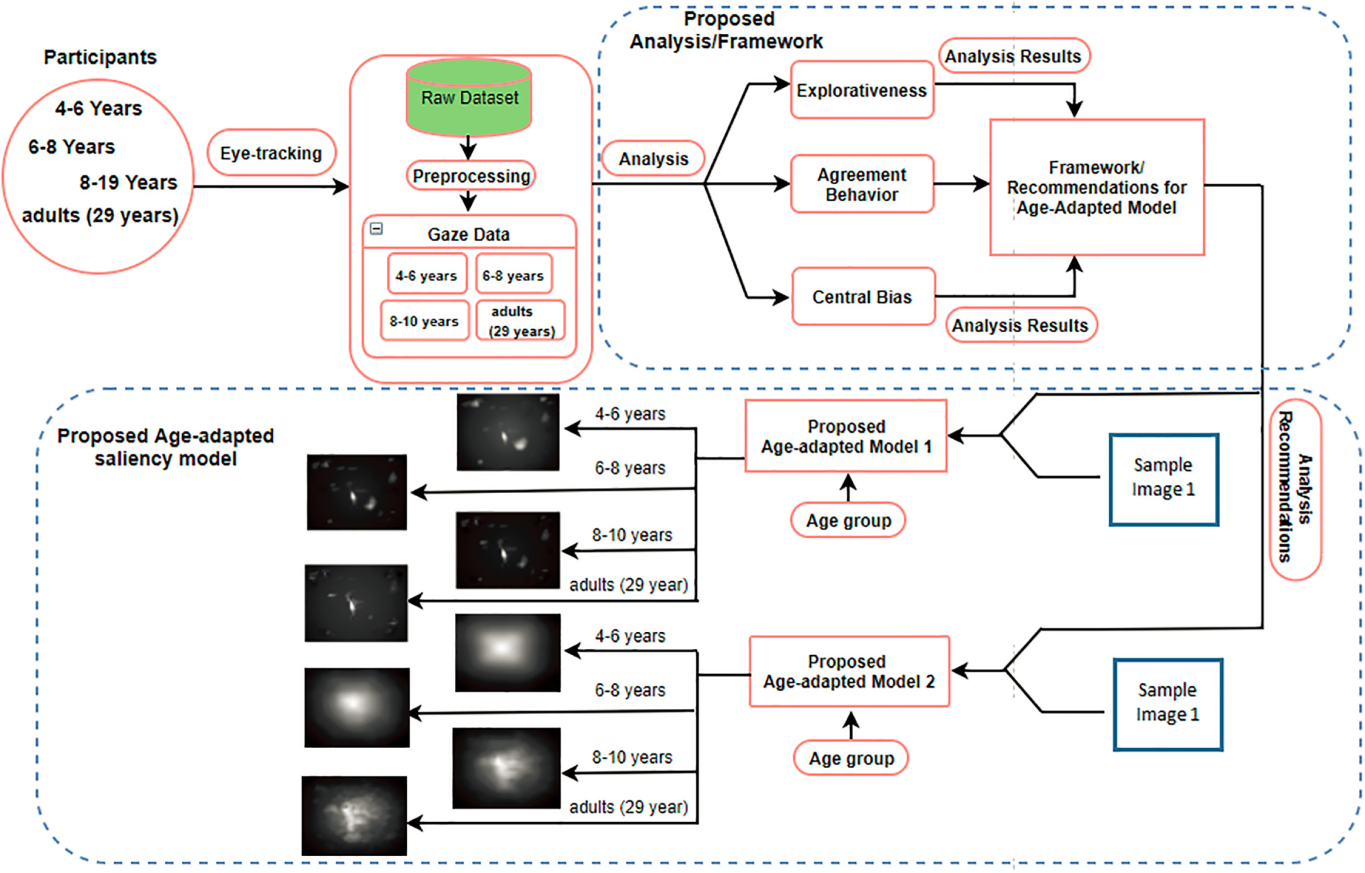
The framework of our proposed study. It consist of two parts, analysis part and proposed age-adapted saliency model as shown in figure, 29 years is mean age of adult observers. The sample image 1 is same for both of the models and it is only representation of the original image.

Our study is divided into two part: the first part consists of quantitative analysis of the age-related differences in fixation landings during scene viewing, and the second part consists of, our proposed age-adapted computational model of saliency prediction based on the analysis results reported in the first part. The framework of the proposed study is shown in Fig 1.

### Visual saliency models

In the last decade, many of computer vision researchers have used psychophysical theories and models of attention such as feature integeration theory (FIT) [5] and Guided search model [27] to create computational models that mimic the adult visual attention system. In this section we briefly review some of these models according to the techniques and/or features they use.

Bottom-up features based models: Itti et al.’s model [3], implemented over FIT theory is one of the most well-known models, where bottom-up features of a scene are extracted in parallel by a set of linear center-surrounded operations similar to the visual receptive field. The graph-based visual saliency model [2] also follows a similar approach to FIT in generating the activation maps of different feature channels at multiple spatial scales. Furthermore, these maps are represented as fully connected graph, where the equilibrium distribution in a Markov chain is treated as the saliency map. However, these models extract features over a fixed spatial scale, and the age-related changes in image feature-related viewing [25] are not considered while generating a final saliency map.

Combination of bottom-up and top-down features: Torralba (2003) [6] and Torralba (2006) [7] proposed a model using a Bayesian framework that integrates the scene context with a bottom-up saliency map.

Similar to the Bayesian framework, the SUN model of saliency prediction [8] combines bottom-up features represented as self-information with top-down information, where top-down information is represented either by Difference of Gaussian (DoG) or independent component analysis (ICA) features extracted from images. A boolean map based model [28] was recently developed based on Gestalt psychological studies [15], and outperformed other state-of-the-art models on saliency related datasets. However, these models do not take into account developmental studies reporting that bottom-up processing is dominating during early development while the influences of top-down processing increase with increasing age [25] [29] [30] [31].

Patch based models: Patch based dissimilarity measures are another line of approach where saliency is estimated in terms of dissimilarity among neighbouring patches. A patch-based saliency estimation method [9] computes the saliency for each patch by measuring the average distance of regional covariance among neighbouring patches. First-order image statistics such as difference of mean value is also incorporated with this algorithm to obtain better results.

Another patch based method [10] was proposed to estimate the saliency of each patches by measuring the spatially-weighted dissimilarity among them, where the image patches were represented in reduced dimensional space by applying principal component analysis (PCA). These models are not suitable for age-adapted prediction of salient locations as the optimal patch size is selected for the highest prediction accuracy over the eye tracking data collected for adult participants only.

Models based on Supervised Learning on Eye tracking datasets: Supervised learning-based models using eye-tracking data collected from adults constitute another technique to build computational models. [4] Proposed a model that simply learns to predict saliency from an eye-tracking dataset containing over 1003 images viewed by 15 adults.

Some of the eye-tracking datasets used for these learning methods are listed in Table 1. It can be seen from the table that the participants of these eye-tracking experiments across all datasets were adults (aged 18 to 50 years).

**Table 1:** Saliency benchmark dataset.

Dataset	Images	Observers	Age	Duration(s)
MIT300 [32]	300	39	18-50	3
FiWI [33]	149	11	21-25	5
NUSEF [34]	758	25	18-35	5
DOVES [35]	101	29	27	5
Toronto [36]	120	20	18-22	4

## Materials and methods

### Eyetracking data

#### Subjects and stimuli

We analyzed the eye-tracking dataset collected in [26]. The eye-tracking data was obtained for 82 observers from different age groups. All observers had normal or corrected-to-normal vision. Participants were assigned to 4 different groups: four-six years, six-eight years, eight-ten years, and adults (mean age, 29 years). We use 4 years, 6 years, 8 years, and adults to refer these groups in order. The study was conducted in conformity with the Code of Ethics of the World Medical Association (Declaration of Helsinki) and approved by the Ethics Committee of the University of Paris Descartes. All participants or their parents in case of children gave written informed consent prior to participation.

The age group assignment was made based on the findings of previous developmental studies suggesting that eye-movement control changes rapidly at the beginning i.e. during childhood, and later more slowly ([37] [38] [39] [23] [24] [19]). These previous findings were also replicated in our analysis results, where we found that the explorativeness, agreement score, and center bias tendency changes significantly during 4 to 10 years of age and gets mature after the age of 8-10 years. These results motivated us to investigate the children age groups in relatively smaller intervals to quantify the significant changes in scene viewing behavior and subsequently reflecting these changes in the age-adapted saliency model.

The experiment was conducted on images of 1024 × 764 pixels. The images were taken from children’s books and movies, and characterized to have eventful backgrounds. Our choice of images might be less interesting for adults than children. However, we decided to use these images since they were suitable for children and they can also be used in eye-gaze study in adults [40], [41], [42]. Paintings [40] and artificial stimulus [41, 42] have been used in these studies to revel the eye movement behavior in adults. These supporting studies provided us an evidence of the suitability of the image type used in the proposed study.

Further, to avoide stimulus related bias and to maintain the motivation of our participants, a segment recognition test was performed during the experiment. In which after the presentation of the image (10s) an image segment was presented at the center of the screen during 5 seconds; in 50% of the cases the image segment was valid. Participants had to determine if the segment was part of the previous scene or not by pressing a button. The results of the task performance reported in [26] suggested the high level of engagement for the selected stimuli for all age groups including adult observers, which also confirms the age appropriateness of our selected stimuli.

#### Apparatus and procedure

The remote eye-tracking system EyeLink 1000 with a sampling rate of 500 Hz was used to measure eye gaze, and provided us with the raw data that was sampled to obtain fixations and saccades. The spatial resolution of eye tracker was below 0.01°, and spatial accuracy more than 0.5°. The random fixations and noise were discarded by processing the raw data by fixation detection algorithm supplied by SR research (EyeLink).

During eye tracking experiment pictures were presented at a distance of 60 cm from a screen at a resolution of 1024 × 728, a five point calibration and validation was performed before starting the viewing task and subjects were asked to explore the scene which was presented for 10 seconds. After this time, the scene was replaced by an image segment and participants had to determine if the segment was part of previous scene or not.

#### Data representation

**Fig 2:**
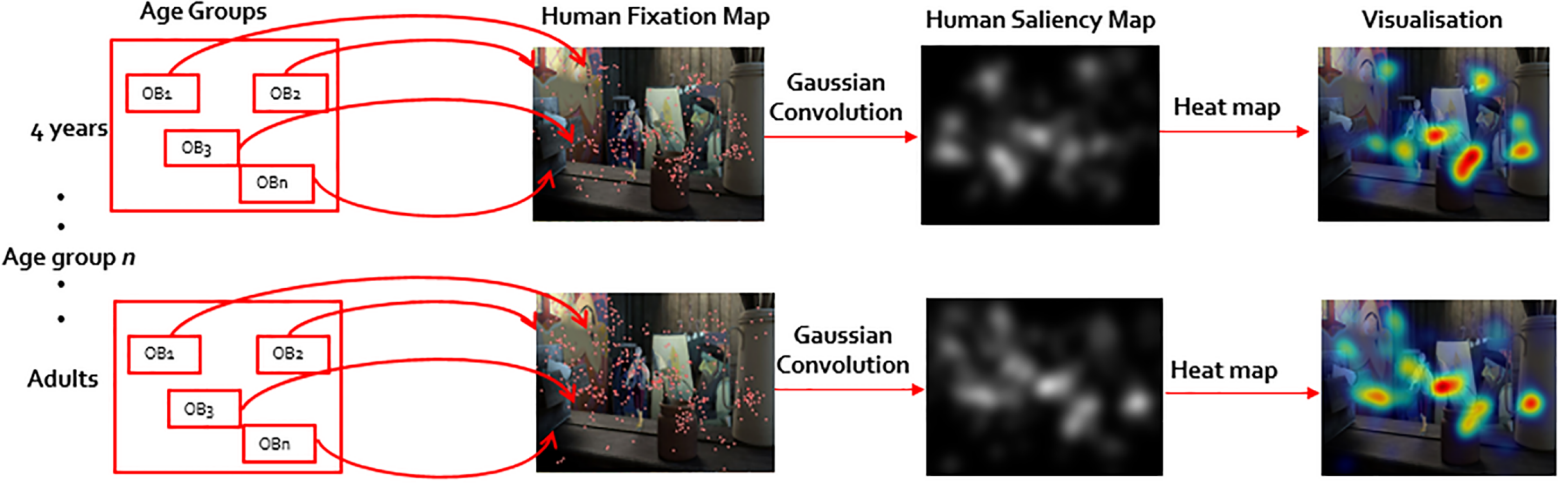
Map generation. The process of generating human fixation map and human saliency maps of an image for age groups. *OB*_*n*_ stands for the *n*^*th*^ observer of an age group. The images used in this figure are similar but not identical to the original image, and is therefore for illustrative purposes only.

For each image fixation landings of all observers were used to generate two maps for different age groups: a human fixation map and a human saliency map. The human fixation map was created as a binary representation of fixation locations, and the human saliency map was obtained by convolving a Gaussian filter across the fixation locations, as in [4]. The visualizations of human fixation and human saliency maps are shown in Fig 2. These maps were used to analyze eye-movement behavior.

### Analysis

To quantify the age-related differences in scene-viewing of observers we selected fixation landing locations as a main attribute to analyse. The reason for this selection relies on the fact that existing saliency models consider fixation location as a key gaze attribute in predicting salient regions. We developed measures to quantify three aspects of viewing behavior: (1) explorativeness, (2) agreement in explored locations within and between age groups, and (3) center bias, each of these contributes to the detailed understanding of how gaze distribution changes for scene viewing with age. The selection of these aspects was based on previous studies in adults showing that the accuracy of saliency prediction in computational models improves when fixation spread, agreement between observers, and center bias tendency are included [4], [8], [43].

The explorativeness was measured using an explorativeness index that indicates the spread of fixation locations. Agreement within and between age groups was estimated by using an agreement score that reflects how well the observers within same age group or of different age groups agrees in terms of explored locations. The last parameter, center bias was which reveals the age-related differences in center bias tendency.

#### Explorativeness

To evaluate eye movement behavior during scene exploration across age groups, we conducted an explorativeness analysis. To quantify the explorativeness of observers in a group we calculated first-order entropy of the human saliency map. The selection of human saliency map for explorativeness study is based on the fact that we observed a human saliency map differs between age groups. For the *i*^*th*^ image of group *g* it is computed as following:

$$\begin{array}{c} H\left(U_{i}^{g}\right)=\sum_{l}h_{U_{i}^{g}}\left(l\right)*log\left(L/h_{U_{i}^{g}}\left(l\right)\right) \end{array}$$ (1)

Where $U_{i}^{g}$ is the human saliency map of the *i*^*th*^ image from all observers in a group *g* for which entropy is calculated and $h_{U_{i}^{g}}\left(l\right)$ is the histogram entry of intensity value *l* in image $U_{i}^{g}$, and *L* is the total number of pixels in $U_{i}^{g}$.

In the context of viewing behavior, a higher entropy corresponds to a more exploratory viewing behavior by the observer, as their saliency points are more scattered in the given scene. Conversely, a lower entropy corresponds to less exploratory behavior. The average behavior of each age group over all images was analyzed based on the average entropy.

#### Agreement analysis

Explorativeness falls short of checking for similarity of explored regions within age groups and between age groups. For instance explorativeness score is unable to answer questions such as: Do observers belonging to the same age group explore the same spatial regions of the image? And is there any agreement among observers in terms of explored regions across age groups? It should be noted that poor agreement of fixation landings between adults and children leads to imprecise prediction when using saliency models that are originally developed for adults. This motivated us to conduct an agreement analysis.

The area under the curve (AUC) is the most commonly used metric in the literature for discrete ground truth saliency maps, and we choose it for our analysis. The AUC-based measure analyzed how well the human saliency map of fixation points of all observers of an age group could be used to find the pooled fixation locations of all observers from the group, as well as observers from different groups. The age group of which the saliency map was used became the source group, and the group for which the fixation locations were being used as target group. Thus, under the intra-age group agreement analysis, the source and target belonged to the same group, and for inter-age group analysis, the source age group was different from the target group.

For this analysis, the human saliency map of the source group was first thresholded to T levels covering different percentages of the most salient areas of the image. To evaluate how well these thresholded maps agreed with the fixation points of the observers in the target group, we then made use of the AUC metrics. This required us to lay down a general formulation of inter-age group metrics—True positive rate (TPR) and false positive rate (FPR) for observers from the source group *g*_*s*_ to find fixation points for observers from the target group *g*_*t*_. The intra-age group metric is a special case of inter-age group metrics, when *g*_*s*_ = *g*_*t*_, i.e., within same group. Thus,

$$\begin{array}{c} TPR_{U_{n}}^{g_{s}g_{t}}\left(I_{i}\right)=\frac{TP_{U_{n}}^{g_{s}g_{t}}\left(I_{i}\right)}{TP_{U_{n}}^{g_{s}g_{t}}\left(I_{i}\right)+FN_{U_{n}}^{g_{s}g_{t}}\left(I_{i}\right)} \end{array}$$ (2)

$$\begin{array}{c} FPR_{U_{n}}^{g_{s}g_{t}}\left(I_{i}\right)=\frac{FP_{U_{n}}^{g_{s}g_{t}}\left(I_{i}\right)}{TP_{U_{n}}^{g_{s}g_{t}}\left(I_{i}\right)+FN_{U_{n}}^{g_{s}g_{t}}\left(I_{i}\right)} \end{array}$$ (3)

Where the TPR for the *i*^*th*^ image is the extent to which the fixation points of observers in group *g*_*t*_ agree to the *n*^*th*^ thresholded saliency map *U*_*n*_ of observers from source group *g*_*s*_. Similarly, FPR deals with non-fixation points that have been considered fixation points. The TPR and FPR for all *T*-thresholded saliency maps of an image were combined into a vector of *T* dimension. The area under the ROC curve plotted between TPR and FPR gave us the AUC-score, and an average of these scores across all stimuli of the dataset provided the agreement score of the group.

**Fig 3:**
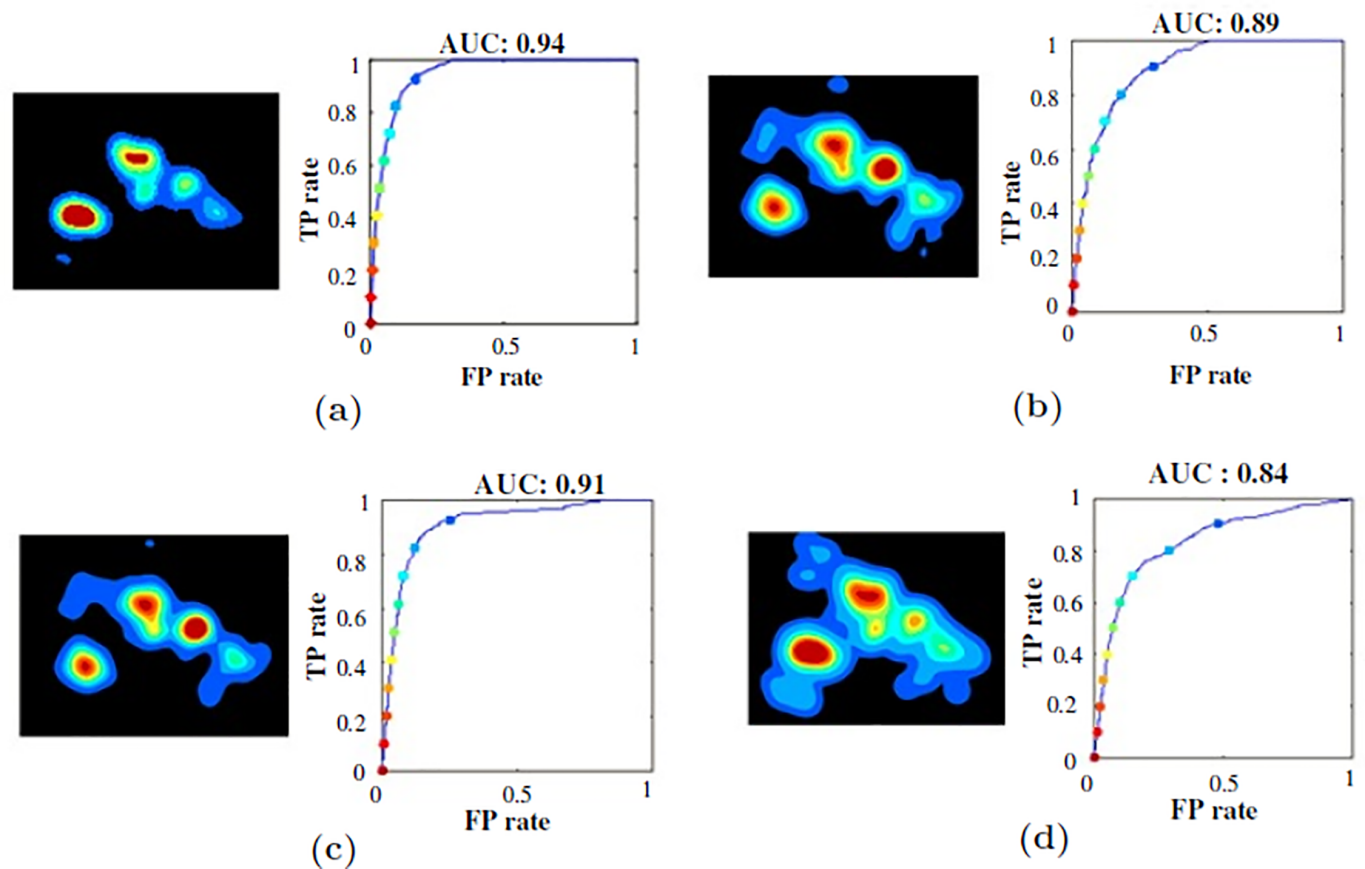
Agreement analysis visualization. The heat map visualizes agreement behavior in predicting the target fixation points by source saliency map and the ROC calculates the quantitative value of the agreement score. (a) Intra-age group: target fixation points of 4 years by source saliency map of 4 years. (b) Intra-age group: target fixation points of adults by source saliency map of adults. (c) Inter-age group: target fixation points of 4 years by source saliency map of adults. (d) Inter-age group: target fixation points of adults by source saliency map of 4 years.

For a given image, agreement score tells us how accurately the fixation locations of all observers in the group were covered under the differently thresholded saliency maps of observers from the same or different age groups. We can visualize the intra-age and inter-age group agreement analysis in Fig 3.

#### Center bias

The term “center bias” has been studied using the eye-tracking techniques, and it reflects the human tendency of looking at the center of a given image [44]. Several studies have established the existence of the center bias, but only a few scholars have considered the center bias in their computational models [4] [11].

The center bias greatly influences our viewing behavior but to the best of our knowledge no study has investigating the age related differences in tendencies toward center bias. In order to reveal differences in center bias across age groups we first computed the center map by taking average of all the saliency maps across age group. Finally, the center bias for each age group is measured by measuring the euclidean distance between the centroid of the center-map and the center pixel of the image. We have also used the center map as a saliency map to predict fixation locations of different age groups. The average prediction performance of the center map for the fixations of different age groups were measured by using AUC matrices developed in agreement analysis section.

We have also investigated the role of contrast bias by following the study reported in [45]. The result suggested that the image contrast plays an important role in gaze landings but the statistical analysis results showed no significant difference between the age groups. Considering that we have only reported age-related changes center bias tendency in this study.

## Results and discussion

### Explorativeness

When participants of 4 years and adults age groups observed the same set of images of our dataset, the set of least explored scenes were found to be different among observers belonging to different age groups. Thus, exploratory behavior depends on the observer‘s age.

**Fig 4:**
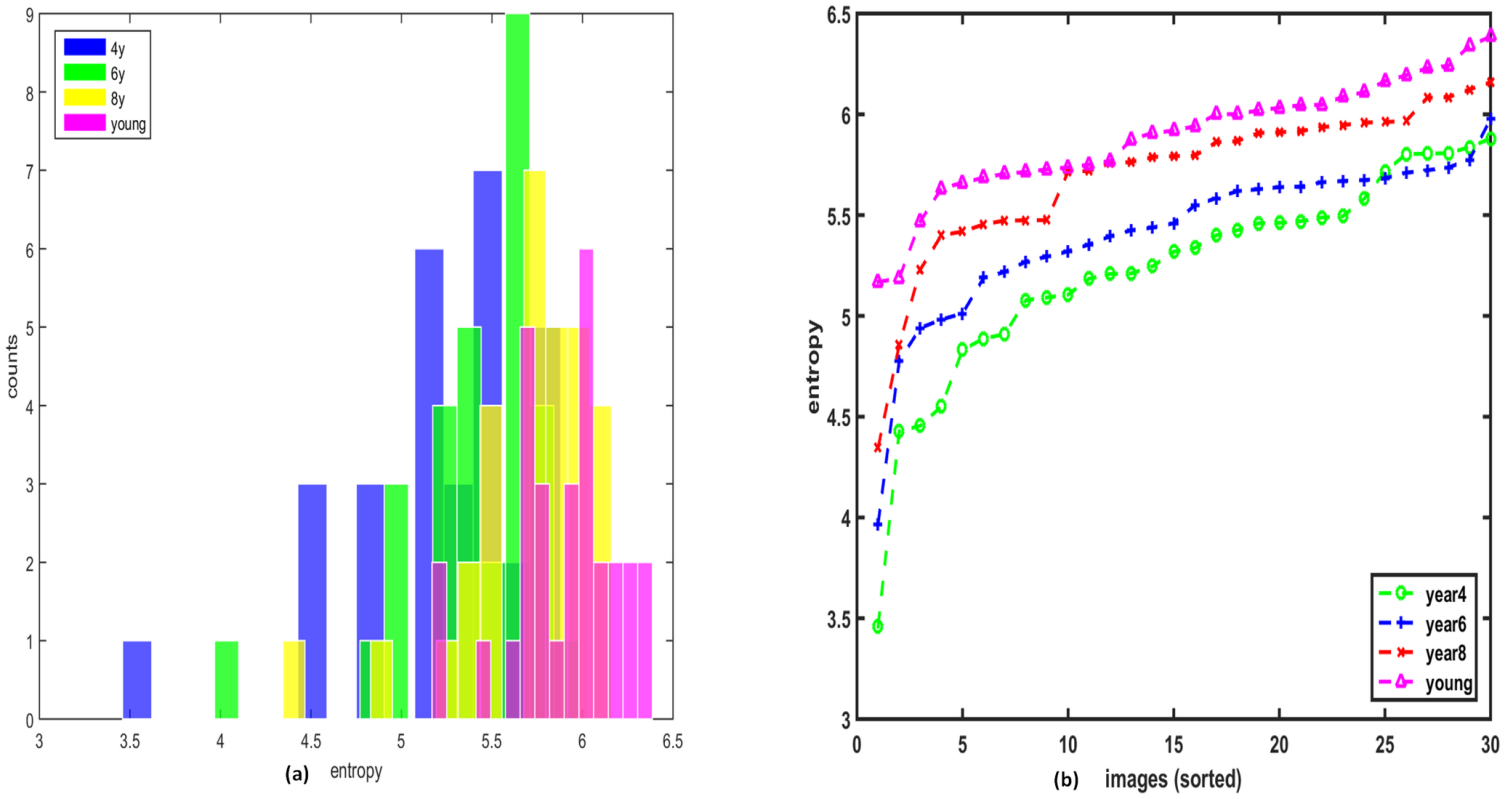
Explorativeness results. (a) The histogram of entropy indicates that there is a shift from left to right for 4 year to adult age group. (b) Entropy plotted in sorted order for different age groups over all the stimuli.

The results of the explorativeness suggested that:

1. Explorativeness increased monotonically with age, *r*(29) = 0.99, *p* < 0.001 (Spearman correlation). This illustrated in Fig 4(b), which plots the entropy of all images for each age group. The histograms of entropy of all images for different age groups are illustrated in Fig 4(a).
2. One-way ANOVA analysis showed that explorativeness varied significantly among the age groups, *F*(3, 29) = 15.8, *p* < 0.001. Bonferroni correction based Post-hoc test indicated that explorativeness scores of 4 and 6 years age groups were significantly lower than the scores of 8 years and adult groups, all *p* < 0.01. However, no difference was found between 8 years and adults age groups, suggesting that from the age of eight years explorativeness behavior is adult-like.

Previously, it has been shown that the spread of the fixation landing i.e. entropy on the saliency map [46] decreases when the image resolution decreases i.e. change in the level of detail responded by changing in the gaze pattern. Thus, our findings suggest that during scene exploration children older than 8 years of age and adults tended to direct their gazes at different level of details in a given scene. On the contrary, being less explorative, children tended to direct their gazes towards fewer details of the scene.

### Agreement analysis

The main results from the intra-age and inter-age group agreement analysis are as follows:

**Intra-age group agreement analysis:**

**Fig 5:**
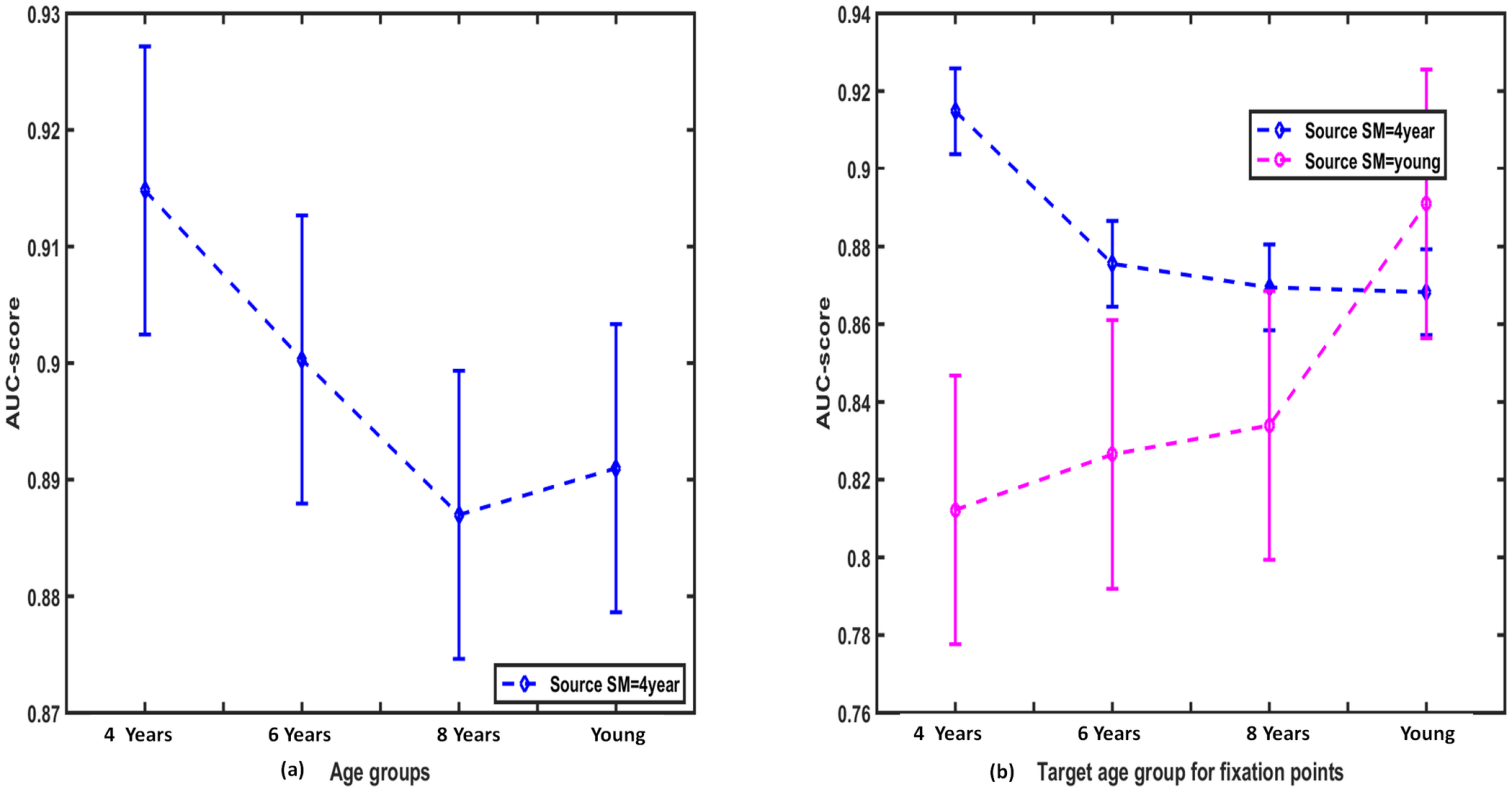
Agreement analysis between observers within age group and across age groups. (a) Intra-age group agreement scores, which reflects that kids agree more in explored locations than younger adults. (b) Agreement analysis results for the source saliency map of 4 years and adults in finding the target fixation of different age groups.

1. There was a negative correlation between intra-age group agreement and observers’ age revealing a high intra-age group agreement between children, *r*(29) = −0.88, *p* < 0.001 (see Fig 5(a)).
2. One-way ANOVA test suggested that the age impacted on agreement score, *F*(3, 29) = 65.8, *p* < 0.01, As shown in Fig 5(a), Bonferroni correction based Post-hoc showed that the average agreement score of the 4 years age group was highest and significantly different from all other age groups (*p* < 0.001). The score started to decrease as observer’s age increased up to 8 years age by showing 8 years old and adults had significantly less intra-age group agreement than 6 years olds, *p* < 0.01.

Similar to the explorativeness results, the agreement score suggested that scene-viewing tendency matures at the age of eight. This can be understood by the fact that 8 years and adults were the most explorative, and there salient regions may not be consistent with one another at higher level of the details.

**Inter-age group agreement analysis:**

1. Table 2 shows that the agreement scores of inter-age group analysis was lower than those of intra-age group analysis for all ages. Thus, it was even more evident that the age has an impact on visual behavior as the same age group maps predicted the fixations more precisely.
2. The most important contribution of the inter-age analysis was that the saliency map of adult subjects showed the poorest performance in predicting the fixation points of the other age groups as shown in Table 2: Agreement score of adults predicting all others (4, 6, 8 years) was significantly less than the agreement scores of diagonal colored boxes of the Table 2 (prediction by same age groups). Spearman’s correlation based post-hoc analysis indicated significant differences in performance of adults predicting 4 year, 6 year, and 8 years than the prediction by the same age-group, *p* < 0.01.

**Table 2:** Agreement score. Average agreement score of human saliency map of observers from the source group in predicting fixation points of target age group.

Source \ Target	4 years	6 years	8 years	adult
4 year	0.9148	0.8756	0.8695	0.8683
6 year	0.8463	0.9003	0.8509	0.8493
8 year	0.8150	0.8269	0.8870	0.8343
adult	0.8122	0.8265	0.8340	0.8910

Thus, ignoring the age factor and using conventional models developed and learned over adults can not give optimal performance for other age groups. This calls for the modification of existing models to make them adapt to age. Fig 5(b) shows the comparison of agreement score for saliency maps of 4 years and adults in finding the target fixations of different age groups.

### Center bias

**Fig 6:**
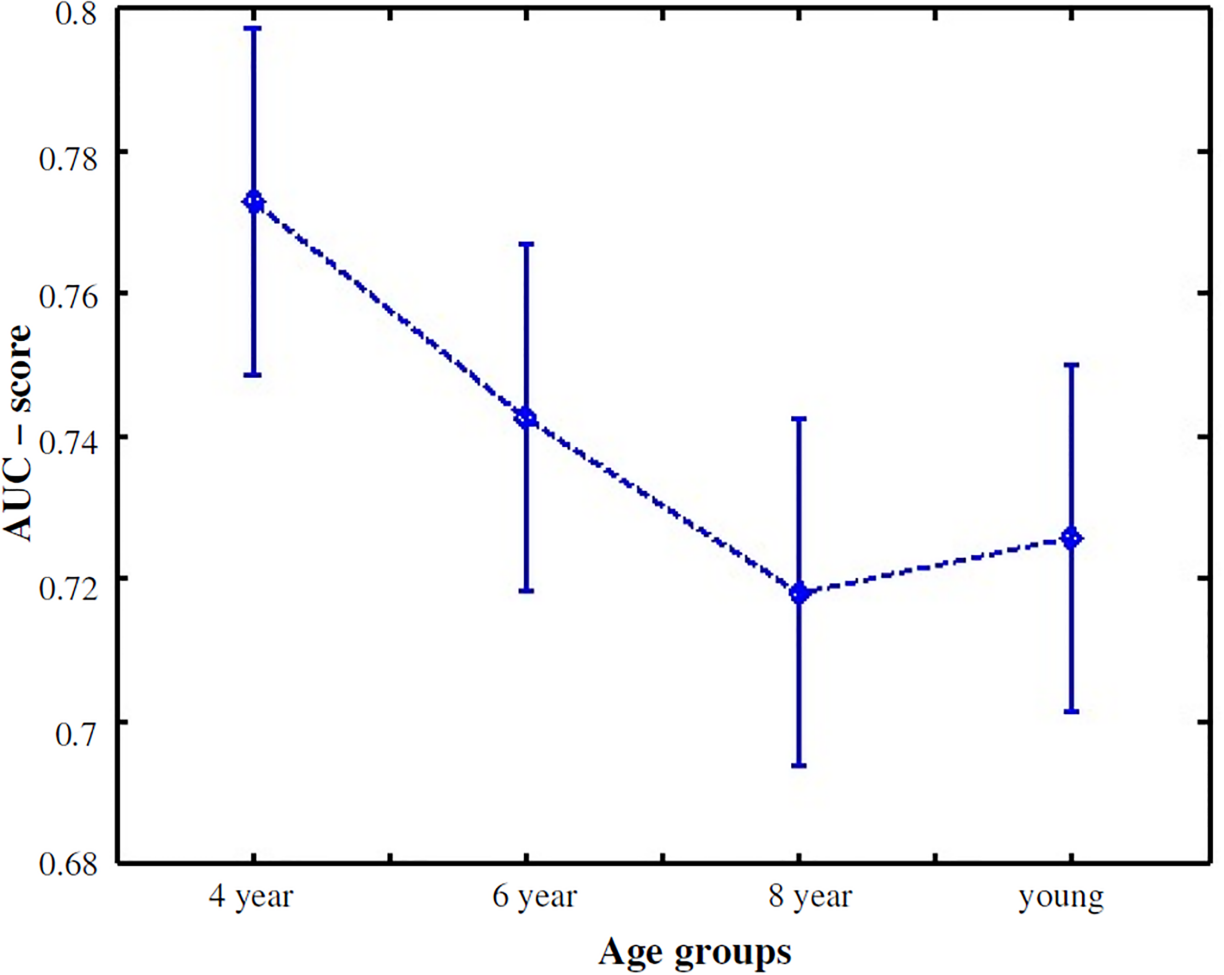
Age-based changes in center bias tendency across age groups.

As shown in Fig 6, age-related differences in bias towards the center map across age groups suggested that the 4 years age group had the highest bias among all age groups. It decreased with increasing age, where adult-like observation behavior was exhibited at 8 years of the age. The results of One way ANOVA analysis indicated the significant age-impact on the center-map bias, *F*(3, 29) = 8.15, *p* < 0.03. Further, post-hoc analysis indicated that both adults and 8 year were significantly different from 4 years and 6 years age groups, *p* < 0.01, similarly, 4 and 6 years also shown significant different with each other *p* < 0.03. The highest euclidean distance for adults suggested the lowest center bias in adults among the age groups (168, 182, 181, and 226 are the euclidean distance in pixels for children, adult and elderly participants).

### A framework for the age-adapted model

Here, we briefly summarize our three main findings that helped us to build the age-adapted computational model:

1. Results of Explorativeness analysis indicate that children (4 and 6 years) exhibited the least explorative behavior among the age groups. Age associated variation in explorativeness indicates that observers of different age groups viewed different levels of detail within a scene. This helped us to choose the scales of features extracted from the images to generate a master saliency map. The features scale selection should be such that they are capable of representing age-based variations at the level of detail of the observer.
2. The intra-age group agreement score was higher than inter-group agreement scores for various combinations of groups. This suggests that while training the model, it is advisable to train the model of a particular age group by using the fixation-map data of the same age group rather than the generalized fixation map data of adults.
3. The magnitude of the center bias was different among age groups. Thus, while including the center bias in age-adapted saliency model, we need to consider the age-related differences in center bias tendency.

## The saliency model adapted to age

As we mentioned before, several computational models for visual saliency have been developed in past work to provide important insights into the underlying mechanisms of the human visual attention system. All existing models predict regions of interest in images by considering the gaze behavior of adults. Thus, these models are optimized to predict fixations of adults, but at the same time, prediction accuracy of these models are not optimal for other age groups. Given a framework of age-adapted saliency model from our analysis result, provides us an opportunity to optimize the prediction performance of the existing models for observers of other age groups as well.

In the proposed work, our age-adapted framework was tested with two types of computational models, the itti’s model [3] and the patch based model [9]. We chose these models carefully in light of the fact that they had different modeling architectures. We verified that the proposed age-adapted framework was generalizable, and could be applied to any type of existing model as, most of them follow the same basic structure with minor variations. Instead of using these models in present form we applied some changes to improve the overall prediction performance of these models. The changes applied to the models were following:

1. The Itti’s model [3], where different visual features are extracted over multiple scales of the input image and a saliency map is obtained by linearly integrating these feature maps into one. We modified the resulting saliency map by applying a weighting factor for the center bias. The proposed age-adapted framework was incorporated with this model by applying the multi-scale feature subset selection with a different set of optimal weights of feature integration learned over our age specific gaze dataset.
2. The aim of the patch-based model is to detect the saliency of the scene based on dissimilarity among neighbouring patches. We modified the existing model by representing a patch with a feature matrix obtained from SVD decomposition. The age-adapted framework was applied by varying patch size and the age-adapted weighting factor for the center bias.

The selection of these models relies on the fact that most of the bottom-up computational models follow this basic structure.

### Age-adapted multi-scale feature subset selection and optimization based model

Most existing bottom-up models follow the basic multi-scale feature selection architecture proposed by Itti et al [3]. In these models, we observe the following basic structure: (a) Basic visual features such as color, intensity, and orientation, are extracted over multiple scales of the image, where each scale represents a different level of detail in the scene. (b) All features are investigated in parallel, to obtain the conspicuity map for each feature channel. (c) These features are integrated to obtain the saliency map.

There are three concerns in developing an age-adapted model over this basic structure of saliency prediction—First, we need to choose the appropriate set of feature scales for different age groups, as our results suggest that different age groups tend to explore different levels of detail in scenes. Second, we need to include the center bias in the proposed model by considering the fact that the strength of the center bias varies with observer age. Third, we need to combine the extracted features over an optimized set of weights for different age groups. This optimization is achieved through a supervised way of learning weights for different age groups.

**(a) Multi-scale feature subset selection: Proposed S**

We used the multi-scale feature extraction technique proposed in the famous Itti et al.’s saliency model [3]. The different scales represented the different levels of detail in scenes, from finer details to coarser object-level details. As stated earlier for more explorative observers, all levels of details were important whereas for the less explorative observers, only coarser-level details were important.

Observers from different age groups showed different levels of explorativeness. Thus, to make our model adapt to age-related differences in scene viewing behavior, we focused on a feature scale selection mechanism, where we identified the subsets of the feature maps that best represented the different levels of details viewed by the observers of different age groups.

We now discuss the steps to extract features for our age-adapted saliency model. For an input image, eight spatial scales were first developed using a Gaussian pyramid. The features were then extracted using the “center-surround” operations with the same settings as in [3] to yield six intensity maps $I_{i}$, 12 color maps—six for ${\text{RG}}_{i}$ and six for ${\text{BY}}_{i}$ each and 24 orientation maps—$O_{i}\left(\theta \right)$ i.e., sets of six maps computed for four orientation *θ* ∈ {0, 45, 90, 135}. The 6 maps for different feature represents different level of detail in scene. The Feature maps were then combined into three “conspicuous maps”, $\bar{I}$ for intensity, $\bar{C}$ for color, and $\bar{O}$ for orientation. However, as stated above, unlike Itti et al.’s model, this point-wise combination was not conducted over all six maps; we also chose subsets of six maps for each age group. The point wise combination of feature map was:

$$\begin{array}{c} \bar{I}=\overset{6}{\underset{i=s}{⨁}}N\left(I_{i}\right) \end{array}$$ (4)

$$\begin{array}{c} \bar{C}=\overset{6}{\underset{i=s}{⨁}}\left[N\left({\text{RG}}_{i}\right)+N\left({\text{BY}}_{i}\right)\right] \end{array}$$ (5)

$$\begin{array}{c} \bar{O}=\sum_{\theta \in \{0,45,90,135\}}\overset{6}{\underset{i=s}{⨁}}N\left(O_{i}\left(\theta \right)\right) \end{array}$$ (6)

where N represents the normalization and *s* is the starting index from where maps were taken.

We developed six cases by varying s to 1, 2, 3, 4, 5, and 6. If s = 1, the subset of feature scale starting from scale 1 (finer) to scale 6 (coarser) had to be combined. Similarly if s = 6, only the feature scale 6 was used. Without using the trend toward explorativeness found in the analysis section, we evaluated the model over all such possible subsets for all groups, and defined the subset for each age that best represented the gaze levels (finer to coarser) of the observers in a given age group.

As shown in prediction results in Table 3, children age groups (4 years, 6 years, and 8 years) are performing better than adults in the existing settings which makes use of all scales (1∼6). However, the prediction accuracy of children age groups are not optimized on the existing scale (1 ∼ 6). The predictive performance of children get optimized if used coarser scales and ignore finer ones (as in Table 3, scale 5 ∼ 6, 4 ∼ 6, and 3 ∼ 6 are optimized scale selection for 4 year, 6 year, and 8 year age groups respectively) while for adults, prediction accuracy was highest if we chose all scales (similar to [3]). It is interesting to note that this result is consistent with our earlier results, i.e., children are less explorative than adults and, hence, require only coarser scales to predict their fixations. Age related differences in center bias tendency was also incorporated in this model by including a differently weighted center-map as explained in age-adapted model for center bias section.

**Table 3:** Average prediction accuracy (AUC-score) by multi-scale feature subset selection. Where as a∼b means from scale a to scale b are selected (Proposed S). Scale 6 is the coarsest level and scale 1 is the finest.

Age \ Scale	1∼6	2∼6	3∼6	4∼6	5∼6	6∼6
4 year	0.7074	0.7180	0.7288	0.7280	**0.7381**	0.7366
6 year	0.6839	0.6940	0.6978	**0.7183**	0.7044	0.7071
8 year	0.6640	0.6655	**0.6722**	0.6664	0.6572	0.6566
adult	**0.6628**	0.6573	0.6541	0.6512	0.6470	0.6495

**(b) Training and Testing: Feature Combination Optimization: Proposed S+I+C**

In this section we proposed another modification in existing models based on our second recommendation reported in a framework for age-adapted model section. The choice of linear integration of feature maps used in previous section was ill-suited because different features contribute differently to the final saliency map. Some state-of-the art models addressed [4] this by learning the optimal weights of feature integration in a supervised manner. These optimal weights are, however, not suitable for our age-adapted mechanism, as they are learned only over eye-tracking data collected for adults. To fit this into our scenario, we learn these optimal weights over features extracted from age-specific subsets of the dataset.

We divided the dataset into a training set with 20 images and a test set with the remaining images. Color, intensity, and orientation features were extracted for the training images. We then selected *P* strongly positive and negative samples, each corresponding to the top and least-rated salient locations of the human saliency map of all observers generated from ground truth eye-tracking data.

Our agreement analysis result suggests that intra-age group fixation point prediction was better than inter-age group performance. In other words, the fixation points of the observers were better predicted by the saliency maps of observers of the same group rather than those of observers of other groups. Thus, the *P* positive and negative samples to be chosen were age group specific, i.e., the positive and negative samples for all age groups were differently chosen for training.

We fixed value *P* to 10; choosing more samples only involved adding redundancy and yielded no performance improvement. For a given set of features and labels (positive and negative samples) for an age group, liblinear SVM was used to learn the model parameters to predict salient locations on the training images. Thus, we obtained model parameters for predefined features over all age groups.

For a given test image, we first collected its features as described in the multi-scale feature selection mechanism, and further predicted saliency values at each pixels as,

$$\begin{array}{c} S_{g}\left(I_{i}\right)=w_{g}X^{T}\left(I_{i}\right)+b_{g} \end{array}$$ (7)

Where *w*_*g*_ and *b*_*g*_ are model parameters learned for each age group *g* and *X*(*I*_*i*_) is feature vector for the *i*^*th*^ test image, this vector is composed of intensity ($\bar{I}$), color ($\bar{C}$), and orientation ($\bar{O}$) features. Based on the saliency values we classified the local pixel as salient or not.

**Fig 7:**
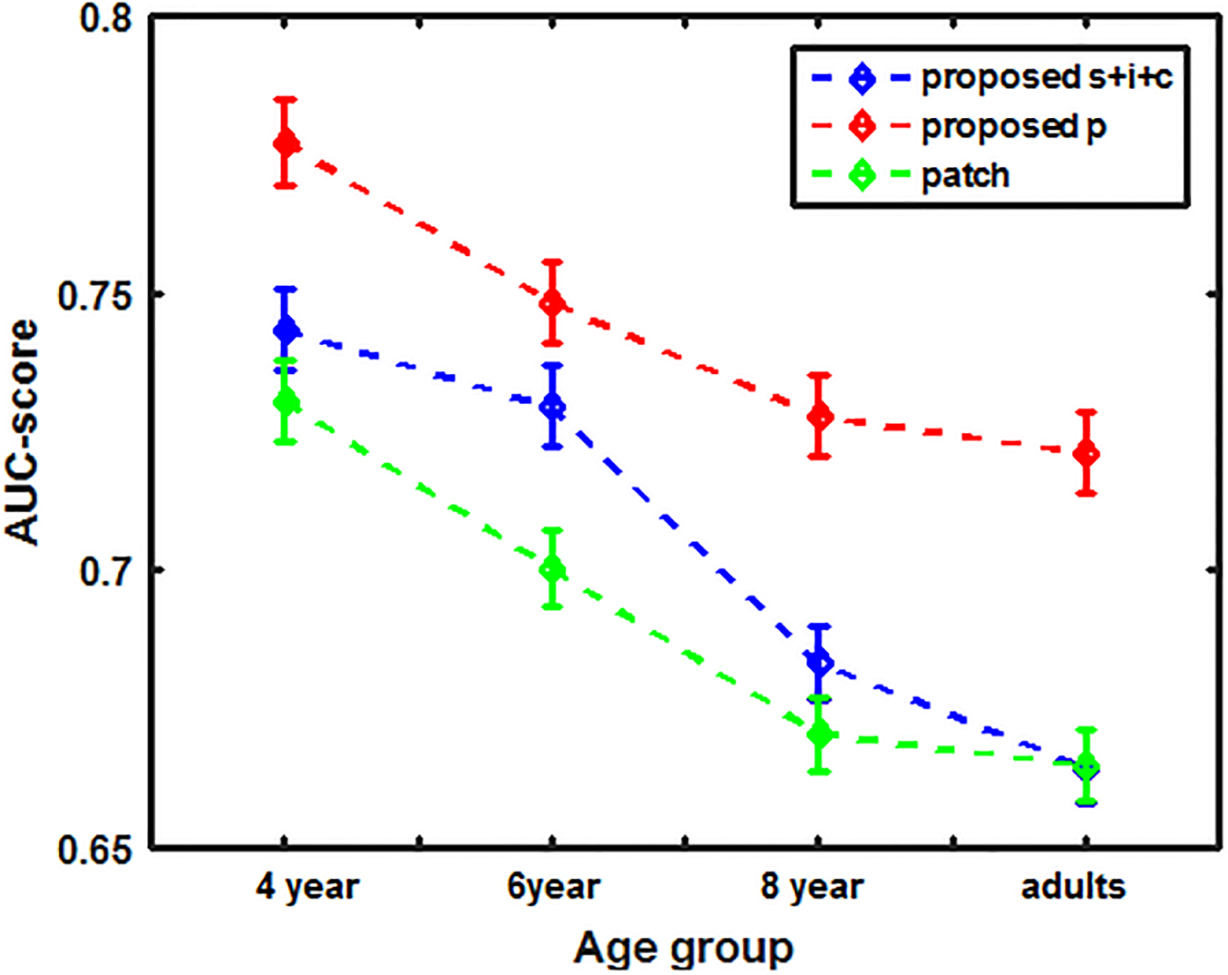
Comparison of age-adapted proposed saliency models with baseline models of computational attention system.

Integrating the feature maps over the optimally set weights learned over the age-specific dataset suggests further improvement in prediction accuracy for all age groups including adults, as shown in Table 4. Age-related differences in center bias tendency were also considered while evaluating the performance of the proposed model. The method of incorporating center bias in the age-adapted model is explained in following section. The improvement in prediction performance for our proposed S and proposed S+I+C model is shown in Fig 7.

**Table 4:** Average prediction accuracy by combining scale based subset selection, nonlinear integration and age-adapted center bias (Proposed S+I+C).

Age \ Scale	1∼6	2∼6	3∼6	4∼6	5∼6	6
4 year	0.7387	0.7409	0.7374	0.7414	**0.7434**	0.7388
6 year	0.7203	0.7218	0.7155	0.7201	**0.7295**	0.7160
8 year	0.6766	**0.6833**	0.6698	0.6590	0.6655	0.6728
adult	**0.6646**	0.6637	0.6613	0.6549	0.6533	0.6585

**(c) Age-adapted model for center bias**

Humans have the tendency to observe at the center of a given scene. This behavior can be incorporated with existing saliency models by simply defining saliency to include weight factor *C*, which is inversely propositional to the distance to the center of the pixel under consideration.

$$\begin{array}{c} C\left(i\right)=1-d\left(c,p_{i}\right)/D \end{array}$$ (8)

where *d*(*c*, *p*_*i*_) is the distance between the pixel under consideration *p*_*i*_ and center pixel *c* and *D* is the maximum distance used as a normalization factor. Further center bias *C*(*i*) is updated based on the results of analysis reflect the age-related variations. *w*_*k*_ *C*(*i*) is the updated center bias weight factor, where *w*_*k*_ is the strength of the center bias tendency for different age groups.

### Age-adapted patch based saliency model: Proposed P

Another approach that we choose to verify the generalizability of our age-adapted framework is the patch-based model for saliency prediction [9]. This technique follows the given basic structure: (a) Image is first divided into patches of the same size. (b) The set of features are extracted from these patches. (c) Finally, the spatial dissimilarity among neighbouring patches is evaluated to generate the saliency map.

As pointed out earlier, we do not use this model as is, but introduce some modifications. For this, we represent different features extracted from a patch by using the subset of eigenvalues obtained after SVD decomposition of the feature matrix. We elaborate this before explaining how to render this newly constructed model age-adapted.

**(i) SVD decomposition based representation of features**

We first construct the feature matrix. The first step in feature matrix construction is to extract non-overlapping patches of size *t* × *t* from a given image I of size *M* × *N*. Thus, the total number of patches $n_{p}=M\times N/t\times t$. Further, each patch is represented by a column vector of features *f*_*i*_, where *i* indexes the patch. *f*_*i*_ is obtained by combining three color of features (L*, a*, b*) and two intesity features (*I*_*x*_, *I*_*y*_). This generates a feature vector for each patch that appears as [*L*_1_, *L*_2_, …., *L*_*t*_, *a*_1_, *a*_2_, …., *a*_*t*_, *b*_1_, *b*_2_, …., *b*_*t*_, *I*_*x*_1__, *I*_*x*_2__, ‥*I*_*x*_*t*__, *I*_*y*_1__, *I*_*y*_2__, …, *I*_*y*_*t*__]. Finally, feature matrix *X*, *X* = [*f*_1_, *f*_2_, …., *f*_*n*_*p*__] for the entire image is obtained by combining the feature vectors of all patches.

Once the feature matrix representation is ready, we generate the covariance matrix representation of feature matrix *X*, *C* = *X*′ *X*^*T*^. Principle component analysis was used to diagonalizes covariance matrix *C* by solving the following eigen vector problem:

$$\begin{array}{c} \lambda V=CV \end{array}$$ (9)

where *V* are the eigen vectors of C and *λ* represents the corresponding eigenvalues. The eigenvectors are ranked in descending order of eigen values. Choosing *d* eigenvectors corresponding to the *d* largest eigenvalues gives us the basis along the directions of maximum variance in features. Thus, the resultant matrix can be represented as *E* = [*V*_1_, *V*_2_, ‥*V*_*d*_]^*T*^.

**(ii) Saliency measurement**

In the final step, saliency can be measured based on the dissimilarity between patches, which can be simply defined as Euclidean distance between patches in reduced dimension.

$$\begin{array}{c} S\left(R_{i}\right)=\omega \left(i\right)\sum_{j=1}^{L}\frac{\sum_{s=1}^{d}\left|x_{si}-x_{sj}\right|}{1+dist(p_{i},p_{j})} \end{array}$$ (10)

where *i*, *j* are the *i*^*th*^ and *j*^*th*^ patches of an image and *ω*(*i*) can be defined as a weight factor to adjust the center bias.

Similarly to the previous model, the age-adapted framework is incorporated into this model by selecting a different subset of patch sizes for different age groups and incorporating the age-adapted center bias. We can select patch sizes from the set {64, 32, 16, 8}, which varies from coarser to finer scale. The result of this model is shown in Table 5. As expected, all scales are suitable for adults, whereas children are more sensitive to fewer scales.

**Table 5:** Average prediction accuracy by proposed patch based method, scale 1, 2, 3, 4 are correspond to a, 16, 8 patch sizes respectively (Proposed P).

Age \ Scale	1∼4	2∼4	3∼4	4∼4
4 year	0.7678	0.7767	**0.7773**	0.7772
6 year	0.7400	**0.7483**	0.7480	0.7482
8 year	0.7195	**0.7279**	0.7272	0.7269
adult	**0.7212**	0.7113	0.7208	0.7188

Table 6 lists the fixation prediction accuracies of some famous existing saliency models executed unaltered for our age specific gaze dataset over observers of different age groups. From Fig 7 and Table 6, it is clear that our modification of Itti’s model and patch-based models that leverage the age-adapted framework outperformed existing models. Our modified age-adapted algorithms improves the prediction performance for adult observers as well. We believe that difference in the fixation prediction accuracies was evidence for the fact that our algorithm not only personalizes saliency models to achieve optimal performance according to the observer’s age group but also improves the prediction performance of adults.

**Table 6:** Comparison table of our proposed (S+I+C and P models) age-adapted models with available computational models of saliency prediction.

Age \ Model	S+I+C	P	Itti’s [3]	GBVS [2]	Judd’s [4]	Patch [9]
4 year	0.7434	**0.7773**	0.6218	0.7184	0.7296	0.7306
6 year	0.7295	**0.7483**	0.6147	0.6969	0.7033	0.7003
8 year	0.6833	**0.7279**	0.6027	0.6722	0.6721	0.6706
adult	0.6646	**0.7212**	0.6062	0.6707	0.6660	0.6649

## Conclusion and discussion

In the first part of the study we analyzed the impact of age on scene viewing of observers belonging to four different age groups from 4 years of age to adults. In the second part, the results of our analyses were used to upgrade two existing models of saliency prediction to make them age-adapted. The analyses were focused on analyzing the age-impact on three basic aspects of gaze distribution behavior: explorativeness, agreement within and between age-groups, and center bias tendency. Selection of these three factors were of particular relevance to the current study as the prior knowledge of gaze distribution behavior of adult observers were used previously in developing more accurate saliency models for adults [43] [4].

A significant impact of age was observed on explorativeness of observers belonging to four different age groups (4, 6, 8 years and adults). Our results showed that explorativeness increased monotonically with age, and adult-like behavior was achieved at the age of 8 years. These results support previous studies comparing explorative performance in young and old adults suggesting that explorativeness change with age [47] [48] and add new evidence about how explorativeness changes during childhood. Our results of higher explorativeness in 8 year-olds and adults agree with previous studies showing that young adults have the highest level of explorativeness. In [49] the authors manipulated the size of the region on which subjects focused their attention to reveal the scope of visual attention between adult and elderly participants. The result showed that elderly participants preferred to attend smaller space than young adults. In the same line [48], [47] and [50] used a task-based study to reveal the possible changes over age in scope of attention. The findings showed that older adults required more time shift the attention to other locations than young adults, suggesting the restricted scope of attention in older adults. However, all of these previous studies investigated the scope of attention in adults and older observers, except the one study reported in [25] which showed the comparable level of exploratory tendency in children of 7-9 years with adults. This result supports our finding that adult-like explorativeness achieved at 8 years of age. Altogether, these findings indicate that explorativeness increases with increasing age and the adult-like explorativeness is reached around 8 year of age.

Our result of concerning the agreement score within the age groups showed that observers of 8 years and adults age group had the lowest agreement with each other. This can be explained by previous study in [43] where the fixation density map of an adult subject was used to predict the fixation density map of other subjects. The result showed a lower inter-similarity score suggesting the lower agreement between adult observers. Comparing the results of explorativeness and agreement analysis, it is interesting to note that the trend followed by the intra-group agreement analysis was opposite to that exhibited by the explorativeness analysis. This makes sense: as explorativeness decreases, observers tends to focus on lesser details of the scene [46], mostly the ones that were the salient areas of an image. This suggests that the fixation points of the observers of the least explorative age group would mostly be consistent with one another, and would be mostly localized at salient locations and, hence, the agreement score would be high.

All the age groups showed significant effect of center bias tendency in scene viewing. However, children of 4 years of age had the highest center bias among different age groups, whereas the adults exhibited lowest center bias tendency for the same set of images. In previous studies center bias tendency has been reported only in adult observers [4] [43], but in our study we provide novel evidence that the strength of the center bias tendency varies across different age groups. One possible explanation is that the center bias is driven by the content of the scene i.e. bottom-up saliency as computed by different saliency models [1, 3–5, 34]. The higher center bias in children observers can be inferred from the previous studies in [25] [26] which found that bottom-up saliency maps dominated more to the fixation landings in children than adult observers, suggesting higher center bias in a children than adults.

Based on the analysis results of our study we concluded that ignoring the age factor and using existing models developed for adults in predicting gaze behavior of other age groups cannot give the optimal prediction accuracy. This motivated us for the development of an age-adapted saliency model. Our results allowed us to develop a more accurate age-adapted model of saliency prediction. The prediction accuracy of the proposed model outperformed the existing stat-of-the-art saliency models for all the age groups including adult observers. Instead of applying age-adapted recommendations directly to [3] [9], we applied several modifications which improved the prediction performance also for adult observers. The present study provides the first systematic approach for quantitatively analyzing the age-related differences in scene viewing behavior with prospective of the development of an age-adapted computational model of visual attention. Additionally, we verified that our proposed framework of age-adapted saliency model is generalizable, and could be applied many other existing model of saliency prediction to make them age-adapted.

## Supporting information

S1 File Saliency map of stimuli-I for 4 years, 6 years, 8 years, and adults age group. (ZIP)

S2 File Saliency map of stimuli-II for 4 years, 6 years, 8 years, and adults age group. (ZIP)

S3 File Saliency map of stimuli-III for 4 years, 6 years, 8 years, and adults age group. (ZIP)

S4 File Saliency map of stimuli-IV three for 4 years, 6 years, 8 years, and adults age group. (ZIP)

S5 File Saliency map of stimuli-V four for 4 years, 6 years, 8 years, and adults age group. (ZIP)

S6 File Saliency map of stimuli-VI five for 4 years, 6 years, 8 years, and adults age group. (ZIP)

S7 File Saliency map of stimuli-VII for 4 years, 6 years, 8 years, and adults age group. (ZIP)

S8 File Saliency map of stimuli-VIII three for 4 years, 6 years, 8 years, and adults age group. (ZIP)

S9 File Saliency map of stimuli-IX four for 4 years, 6 years, 8 years, and adults age group. (ZIP)

S10 File Saliency map of stimuli-X five for 4 years, 6 years, 8 years, and adults age group. (ZIP)

## References

[1] Zhang L, Yang L, Luo T. Unified saliency detection model using color and texture features. PloS one. 2016;11(2):e0149328 doi: 10.1371/journal.pone.0149328 26889826 10.1371/journal.pone.0149328 PMC4758633

[2] Harel J, Koch C, Perona P. Graph-based visual saliency. In: Advances in neural information processing systems; 2007. p. 545–552.

[3] Itti L, Koch C, Niebur E. A model of saliency-based visual attention for rapid scene analysis. IEEE Transactions on pattern analysis and machine intelligence. 1998;20(11):1254–1259. doi: 10.1109/34.730558

[4] Judd T, Ehinger K, Durand F, Torralba A. Learning to predict where humans look. In: Computer Vision, 2009 IEEE 12th international conference on. IEEE; 2009. p. 2106–2113.

[5] Treisman A. The perception of features and objects in Attention: Selection, Awareness and Control; 1993.

[6] Torralba A. Modeling global scene factors in attention. JOSA A. 2003;20(7):1407–1418. doi: 10.1364/JOSAA.20.001407 12868645 10.1364/josaa.20.001407

[7] Torralba A, Oliva A, Castelhano MS, Henderson JM. Contextual guidance of eye movements and attention in real-world scenes: the role of global features in object search. Psychological review. 2006;113(4):766 doi: 10.1037/0033-295X.113.4.766 17014302 10.1037/0033-295X.113.4.766

[8] Zhang L, Tong MH, Marks TK, Shan H, Cottrell GW. SUN: A Bayesian framework for saliency using natural statistics. Journal of vision. 2008;8(7):32–32. doi: 10.1167/8.7.32 19146264 10.1167/8.7.32 PMC7360059

[9] Duan L, Wu C, Miao J, Qing L, Fu Y. Visual saliency detection by spatially weighted dissimilarity. In: Computer Vision and Pattern Recognition (CVPR), 2011 IEEE Conference on. IEEE; 2011. p. 473–480.

[10] Erdem E, Erdem A. Visual saliency estimation by nonlinearly integrating features using region covariances. Journal of vision. 2013;13(4):11–11. doi: 10.1167/13.4.11 23509407 10.1167/13.4.11

[11] Gautier J, Le Meur O. A time-dependent saliency model combining center and depth biases for 2D and 3D viewing conditions. Cognitive Computation. 2012;4(2):141–156. doi: 10.1007/s12559-012-9138-3

[12] Borenstein E, Ullman S. Combined top-down/bottom-up segmentation. IEEE Transactions on pattern analysis and machine intelligence. 2008;30(12):2109–2125. doi: 10.1109/TPAMI.2007.70840 18988946 10.1109/TPAMI.2007.70840

[13] Tian H, Fang Y, Zhao Y, Lin W, Ni R, Zhu Z. Salient region detection by fusing bottom-up and top-down features extracted from a single image. IEEE Transactions on Image processing. 2014;23(10):4389–4398. doi: 10.1109/TIP.2014.2350914 25163061 10.1109/TIP.2014.2350914

[14] Rao RP, Zelinsky GJ, Hayhoe MM, Ballard DH. Eye movements in iconic visual search. Vision research. 2002;42(11):1447–1463. doi: 10.1016/S0042-6989(02)00040-8 12044751 10.1016/s0042-6989(02)00040-8

[15] Kohler W. Gestalt Psychology (1929). New York, NY: Liveright 1947;.

[16] Balkenius C. Attention, habituation and conditioning: Toward a computational model. Cognitive Science Quarterly. 2000;1(2):171–214.

[17] Taylor J, Fragopanagos N. Modelling the interaction of attention and emotion. In: Neural Networks, 2005. IJCNN’05. Proceedings. 2005 IEEE International Joint Conference on. vol. 3. IEEE; 2005. p. 1663–1668.

[18] Johnson SP. Development of visual perception. Wiley Interdisciplinary Reviews: Cognitive Science. 2011;2(5):515–528. 26302303 10.1002/wcs.128

[19] Luna B, Velanova K, Geier CF. Development of eye-movement control. Brain and cognition. 2008;68(3):293–308. doi: 10.1016/j.bandc.2008.08.019 18938009 10.1016/j.bandc.2008.08.019 PMC2731686

[20] Chandna A. Natural history of the development of visual acuity in infants. Eye. 1991;5(1):20 doi: 10.1038/eye.1991.4 2060665 10.1038/eye.1991.4

[21] Roucoux A, Culee C, Roucoux M. Development of fixation and pursuit eye movements in human infants. Behavioural brain research. 1983;10(1):133–139. doi: 10.1016/0166-4328(83)90159-6 6639721 10.1016/0166-4328(83)90159-6

[22] Fukushima J, Hatta T, Fukushima K. Development of voluntary control of saccadic eye movements: I. Age-related changes in normal children. Brain and Development. 2000;22(3):173–180. doi: 10.1016/S0387-7604(00)00101-7 10814900 10.1016/s0387-7604(00)00101-7

[23] Irving EL, Steinbach MJ, Lillakas L, Babu RJ, Hutchings N. Horizontal saccade dynamics across the human life span. Investigative ophthalmology & visual science. 2006;47(6):2478–2484. doi: 10.1167/iovs.05-1311 16723459 10.1167/iovs.05-1311

[24] Klein C, Foerster F. Development of prosaccade and antisaccade task performance in participants aged 6 to 26 years. Psychophysiology. 2001;38(2):179–189. doi: 10.1111/1469-8986.3820179 11347863

[25] Açık A, Sarwary A, Schultze-Kraft R, Onat S, König P. Developmental changes in natural viewing behavior: bottom-up and top-down differences between children, young adults and older adults. Frontiers in psychology. 2010;1:207 doi: 10.3389/fpsyg.2010.00207 21833263 10.3389/fpsyg.2010.00207 PMC3153813

[26] Helo A, Pannasch S, Sirri L, Rämä P. The maturation of eye movement behavior: Scene viewing characteristics in children and adults. Vision research. 2014;103:83–91. doi: 10.1016/j.visres.2014.08.006 25152319 10.1016/j.visres.2014.08.006

[27] Wolfe JM. Guided search 2.0 a revised model of visual search. Psychonomic bulletin & review. 1994;1(2):202–238. doi: 10.3758/BF03200774 24203471 10.3758/BF03200774

[28] Zhang J, Sclaroff S. Saliency detection: A boolean map approach. In: Computer Vision (ICCV), 2013 IEEE International Conference on. IEEE; 2013. p. 153–160.

[29] Castelhano MS, Mack ML, Henderson JM. Viewing task influences eye movement control during active scene perception. Journal of vision. 2009;9(3):6–6. doi: 10.1167/9.3.6 19757945 10.1167/9.3.6

[30] Mills M, Hollingworth A, Van der Stigchel S, Hoffman L, Dodd MD. Examining the influence of task set on eye movements and fixations. Journal of vision. 2011;11(8):17–17. doi: 10.1167/11.8.17 21799023 10.1167/11.8.17 PMC3163592

[31] Tatler BW, Vincent BT. Systematic tendencies in scene viewing. Journal of Eye Movement Research. 2008;2(2).

[32] Judd T, Durand F, Torralba A. A benchmark of computational models of saliency to predict human fixations. 2012;.

[33] Shen C, Zhao Q. Webpage saliency In: European conference on computer vision. Springer; 2014 p. 33–46.

[34] Ma KT, Sim T, Kankanhalli M. VIP: A unifying framework for computational eye-gaze research. In: International Workshop on Human Behavior Understanding. Springer; 2013. p. 209–222.

[35] Van Der Linde I, Rajashekar U, Bovik AC, Cormack LK. DOVES: a database of visual eye movements. Spatial vision. 2009;22(2):161–177. doi: 10.1163/156856809787465636 19228456 10.1163/156856809787465636

[36] Bruce N, Tsotsos J. Attention based on information maximization. Journal of Vision. 2007;7(9):950–950. doi: 10.1167/7.9.950

[37] Aring E, Grönlund MA, Hellström A, Ygge J. Visual fixation development in children. Graefe’s Archive for Clinical and Experimental Ophthalmology. 2007;245(11):1659–1665. doi: 10.1007/s00417-007-0585-6 17453232 10.1007/s00417-007-0585-6

[38] Fukushima J, Akao T, Kurkin S, Kaneko CR, Fukushima K. The vestibular-related frontal cortex and its role in smooth-pursuit eye movements and vestibular-pursuit interactions. Journal of Vestibular Research. 2006;16(1, 2):1–22. 16917164 PMC1761700

[39] Ygge J, Aring E, Han Y, Bolzani R, HellstrÖm A. Fixation stability in normal children. Annals of the New York Academy of Sciences. 2005;1039(1):480–483. doi: 10.1196/annals.1325.049 15827004 10.1196/annals.1325.049

[40] Pannasch S, Schulz J, Velichkovsky BM. On the control of visual fixation durations in free viewing of complex images. Attention, Perception, & Psychophysics. 2011;73(4):1120–1132. doi: 10.3758/s13414-011-0090-1 10.3758/s13414-011-0090-1 21271317

[41] Madden DJ, Whiting WL. Age-related changes in visual attention. Recent advances in psychology and aging. 2004; p. 41–88.

[42] Karatekin C. Eye tracking studies of normative and atypical development. Developmental review. 2007;27(3):283–348. doi: 10.1016/j.dr.2007.06.006

[43] Ma CY, Hang HM. Learning-based saliency model with depth information. Journal of vision. 2015;15(6):19–19. doi: 10.1167/15.6.19 10.1167/15.6.19 26024466

[44] Tatler BW. The central fixation bias in scene viewing: Selecting an optimal viewing position independently of motor biases and image feature distributions. Journal of vision. 2007;7(14):4–4. doi: 10.1167/7.14.4 18217799 10.1167/7.14.4

[45] Cheng MM, Mitra NJ, Huang X, Torr PH, Hu SM. Global contrast based salient region detection. IEEE Transactions on Pattern Analysis and Machine Intelligence. 2015;37(3):569–582. doi: 10.1109/TPAMI.2014.2345401 26353262 10.1109/TPAMI.2014.2345401

[46] Judd T, Durand F, Torralba A. Fixations on low-resolution images. Journal of Vision. 2011;11(4):14–14. doi: 10.1167/11.4.14 21518823 10.1167/11.4.14

[47] Hartley AA, Kieley J, Mckenzie CR. Allocation of visual attention in younger and older adults. Perception & Psychophysics. 1992;52(2):175–185. doi: 10.3758/BF03206771 1508625 10.3758/bf03206771

[48] LaBerge D. Spatial extent of attention to letters and words. Journal of Experimental Psychology: Human Perception and Performance. 1983;9(3):371 6223977 10.1037//0096-1523.9.3.371

[49] Kosslyn SM, Brown HD, Dror IE. Aging and the scope of visual attention. Gerontology. 1999;45(2):102–109. doi: 10.1159/000022071 9933733 10.1159/000022071

[50] Cerella J, Poon L. Age and parafoveal sensitivity. In: meeting of the Gerontological Society of America; 1981.

