# Supplementary figures and images for: Gaze distribution analysis and saliency prediction across age groups
Source: PLoS One. 2018 Feb 23;13(2):e0193149. doi: 10.1371/journal.pone.0193149 (PMC5825055; doi:10.1371/journal.pone.0193149)

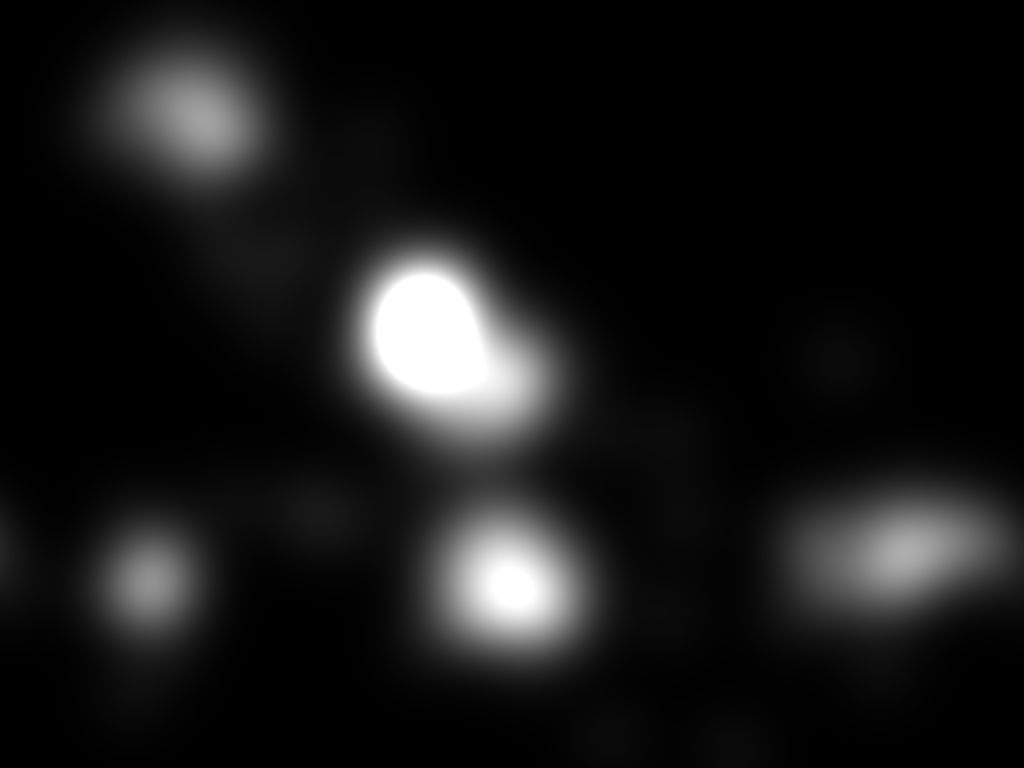

Supplement: S1 File — (ZIP) [file pone.0193149.s001.zip › S1/S1_4year.png]

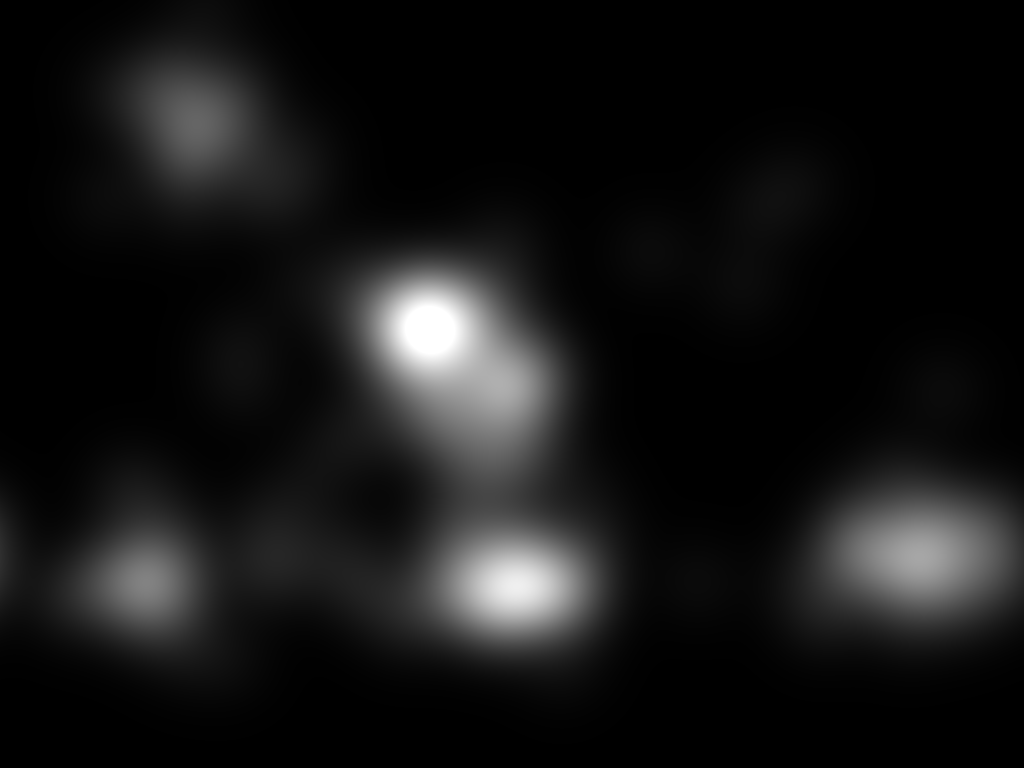

Supplement: S1 File — (ZIP) [file pone.0193149.s001.zip › S1/S1_6year.png]

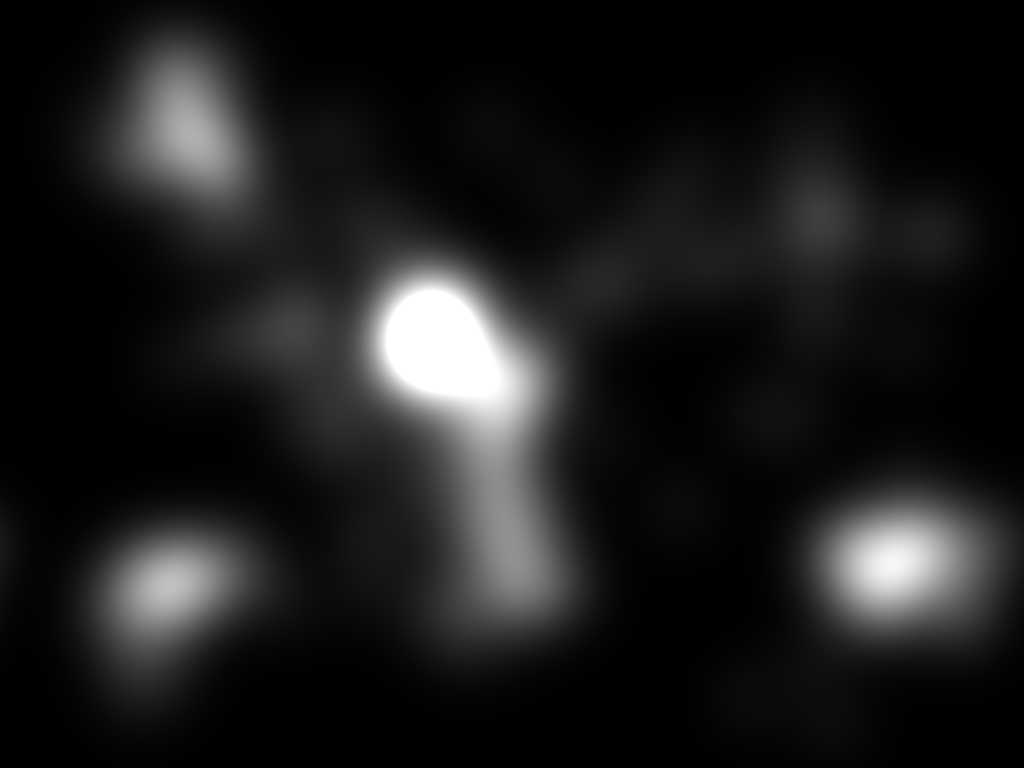

Supplement: S1 File — (ZIP) [file pone.0193149.s001.zip › S1/S1_8year.png]

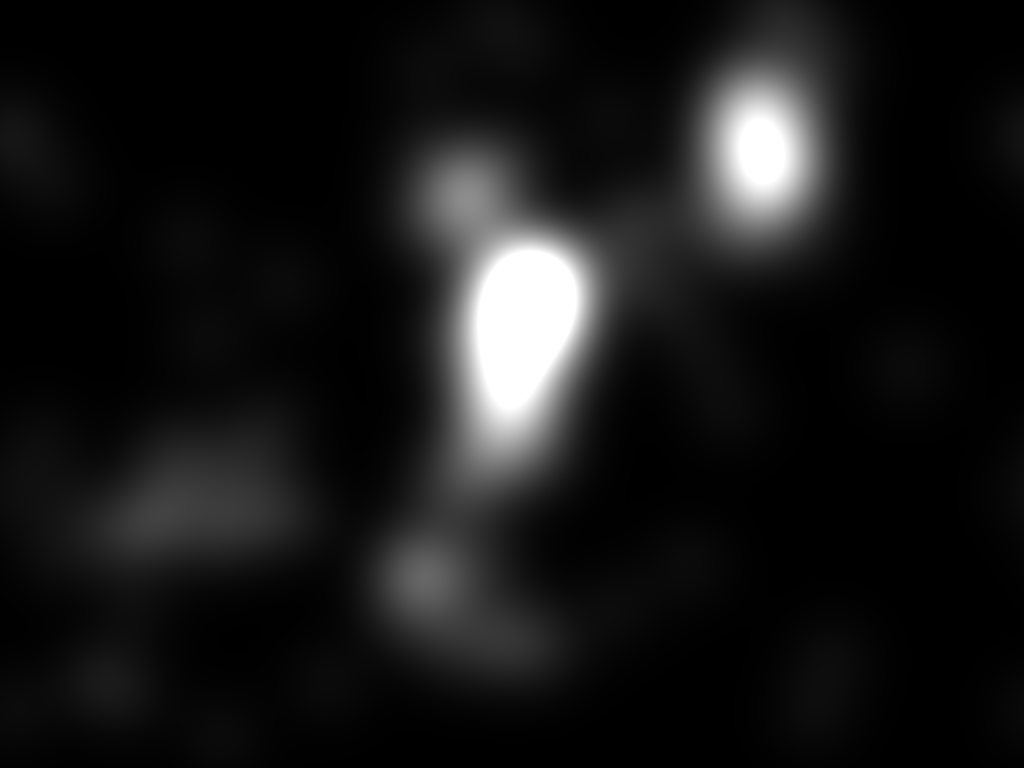

Supplement: S2 File — (ZIP) [file pone.0193149.s002.zip › S2/S2_4year.png]

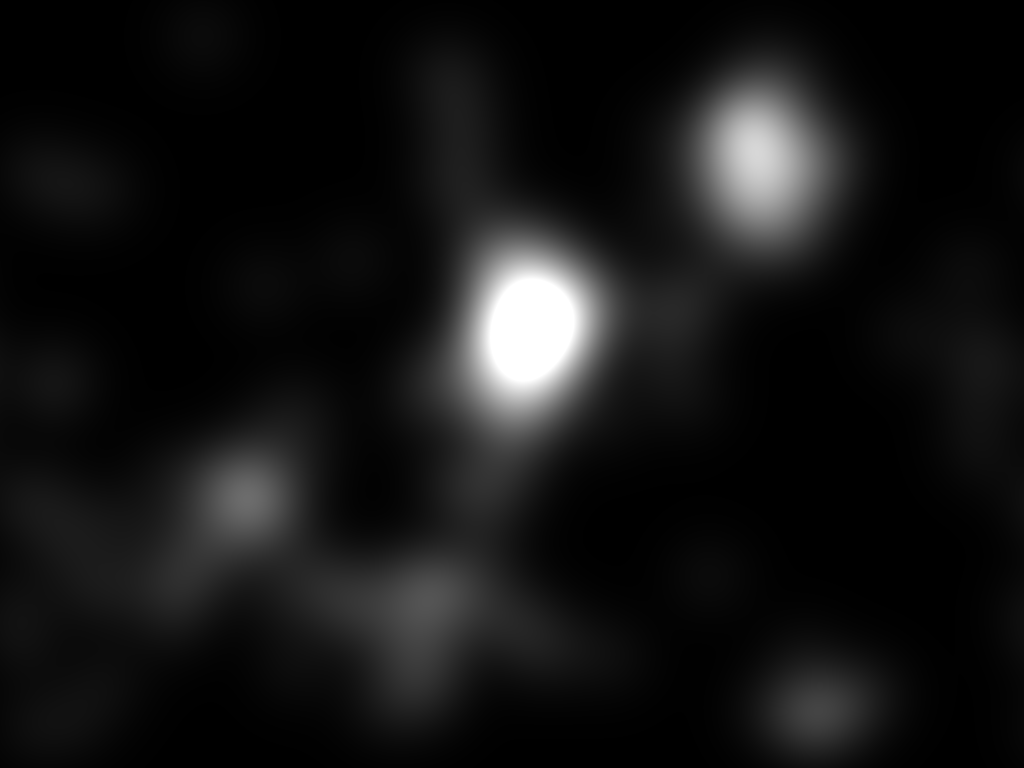

Supplement: S2 File — (ZIP) [file pone.0193149.s002.zip › S2/S2_6year.png]

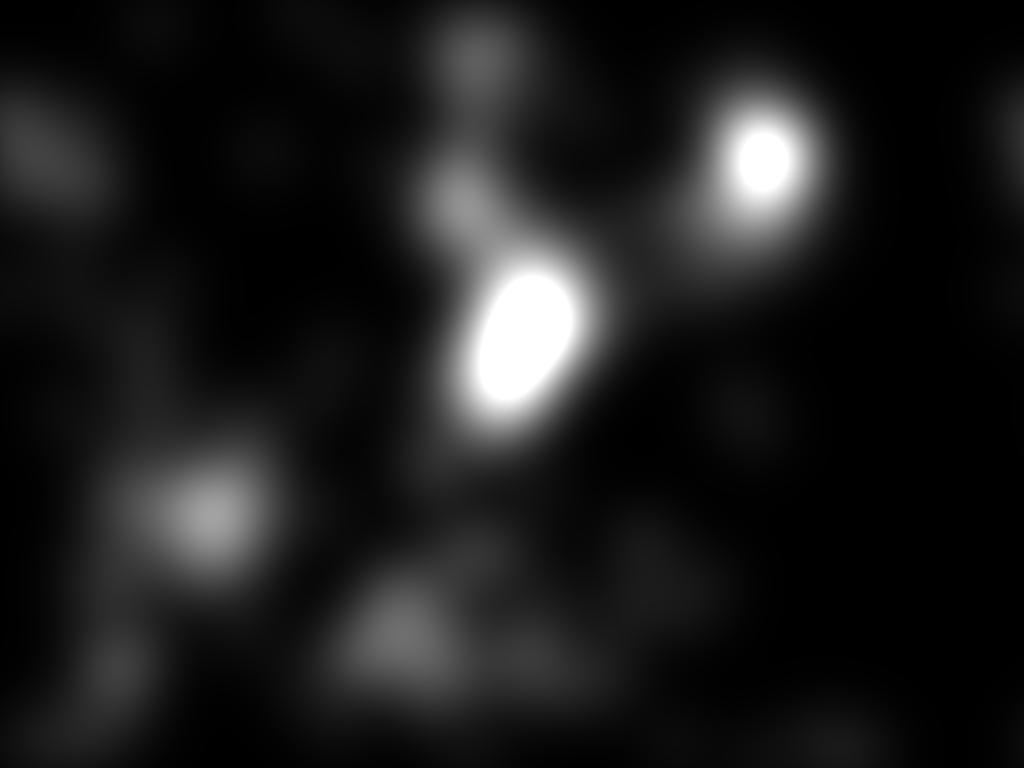

Supplement: S2 File — (ZIP) [file pone.0193149.s002.zip › S2/S2_8year.png]

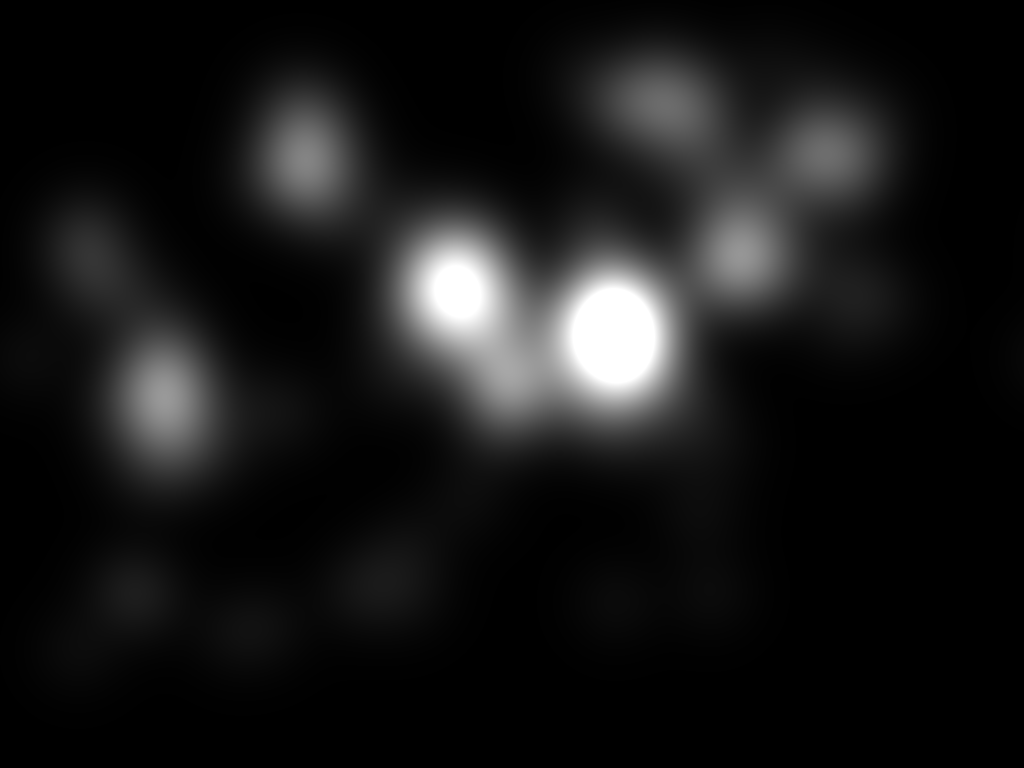

Supplement: S3 File — (ZIP) [file pone.0193149.s003.zip › S3/S3_4year.png]

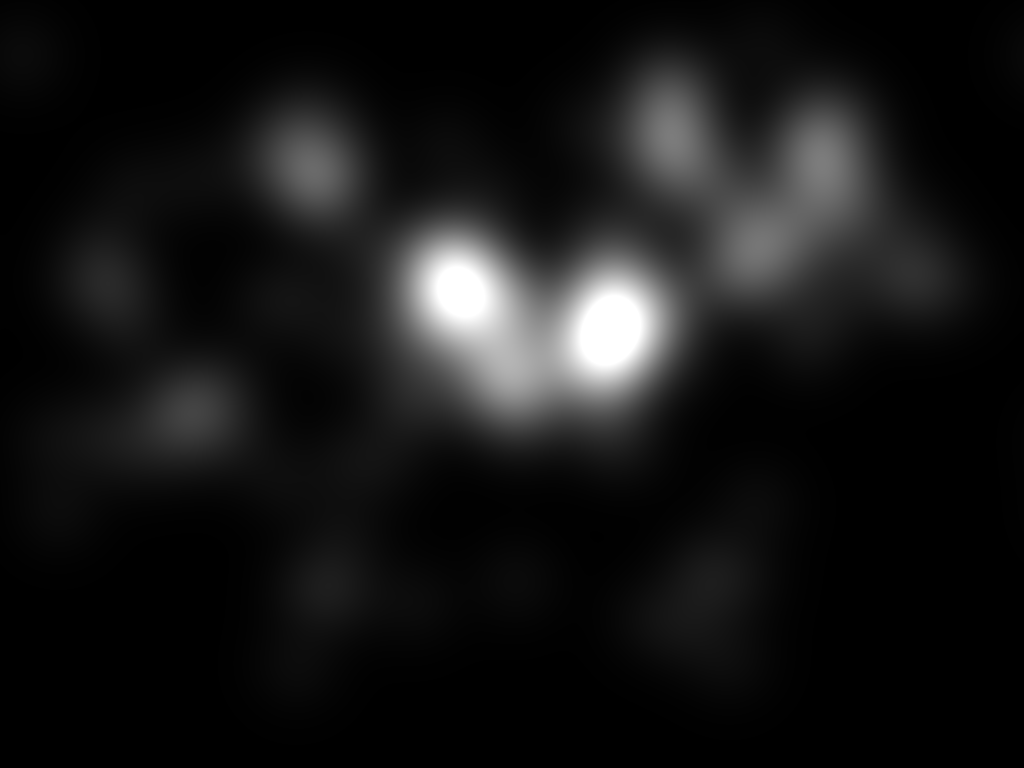

Supplement: S3 File — (ZIP) [file pone.0193149.s003.zip › S3/S3_6year.png]

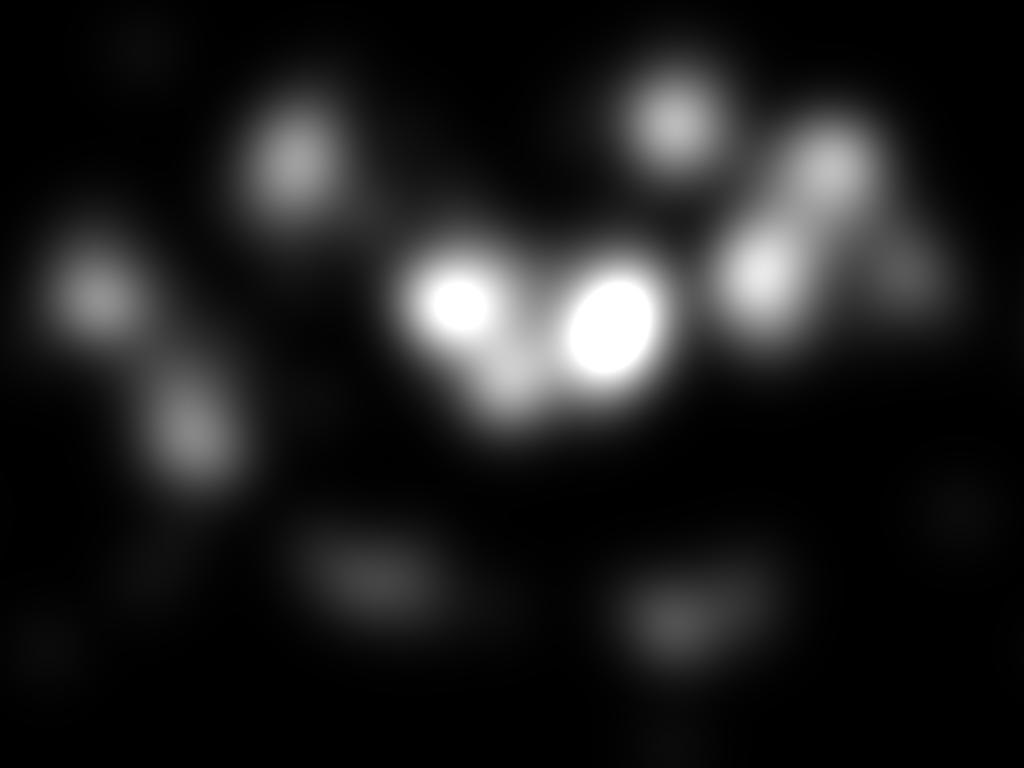

Supplement: S3 File — (ZIP) [file pone.0193149.s003.zip › S3/S3_8year.png]

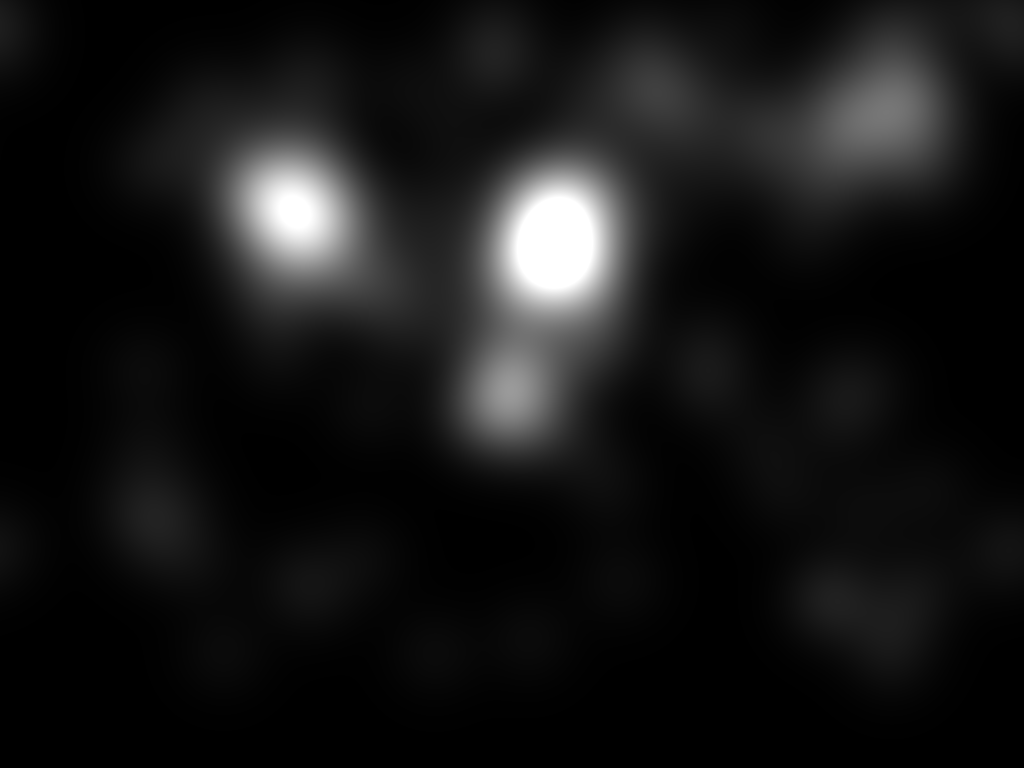

Supplement: S4 File — (ZIP) [file pone.0193149.s004.zip › S4/S4_4year.png]

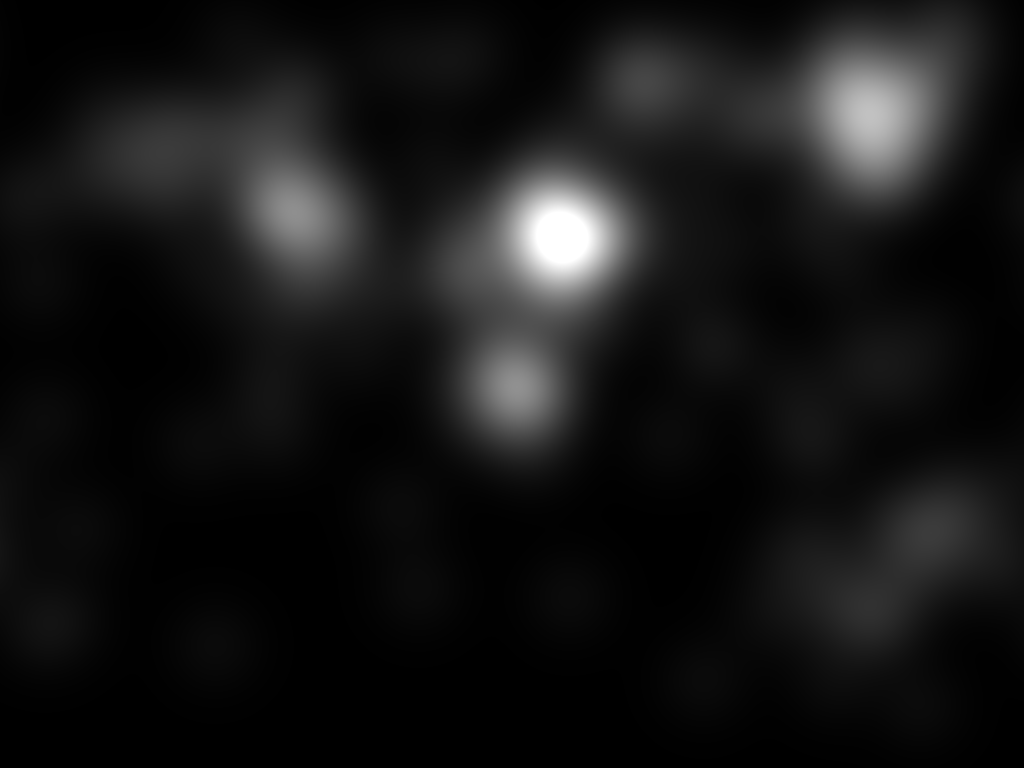

Supplement: S4 File — (ZIP) [file pone.0193149.s004.zip › S4/S4_6year.png]

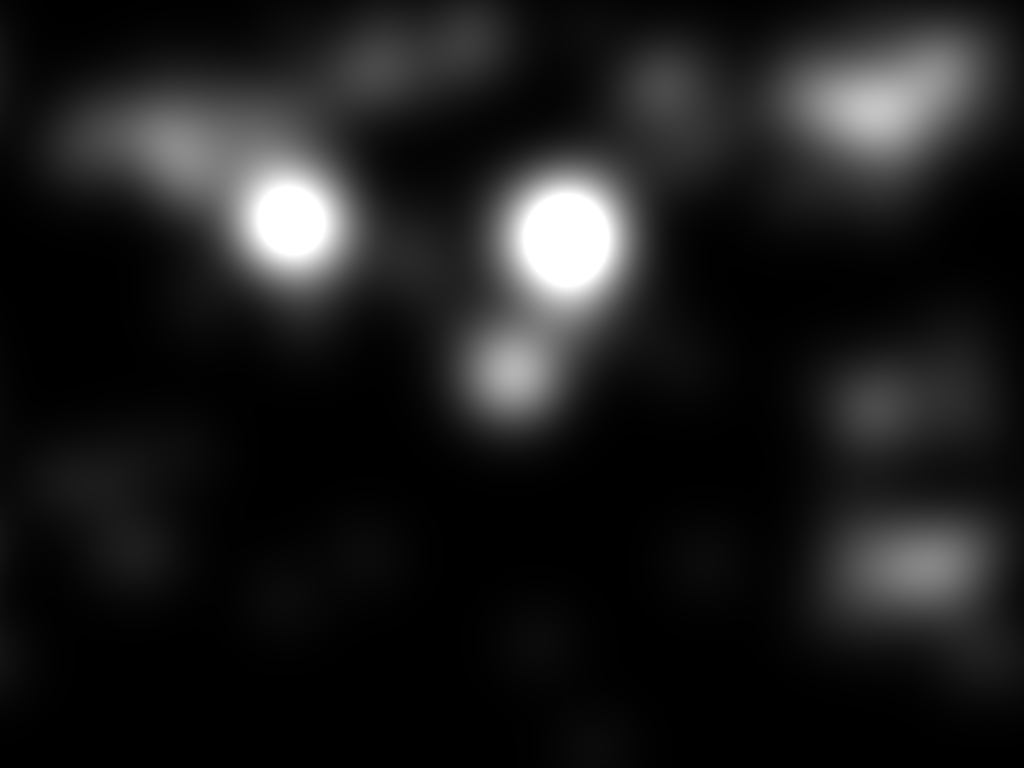

Supplement: S4 File — (ZIP) [file pone.0193149.s004.zip › S4/S4_8year.png]

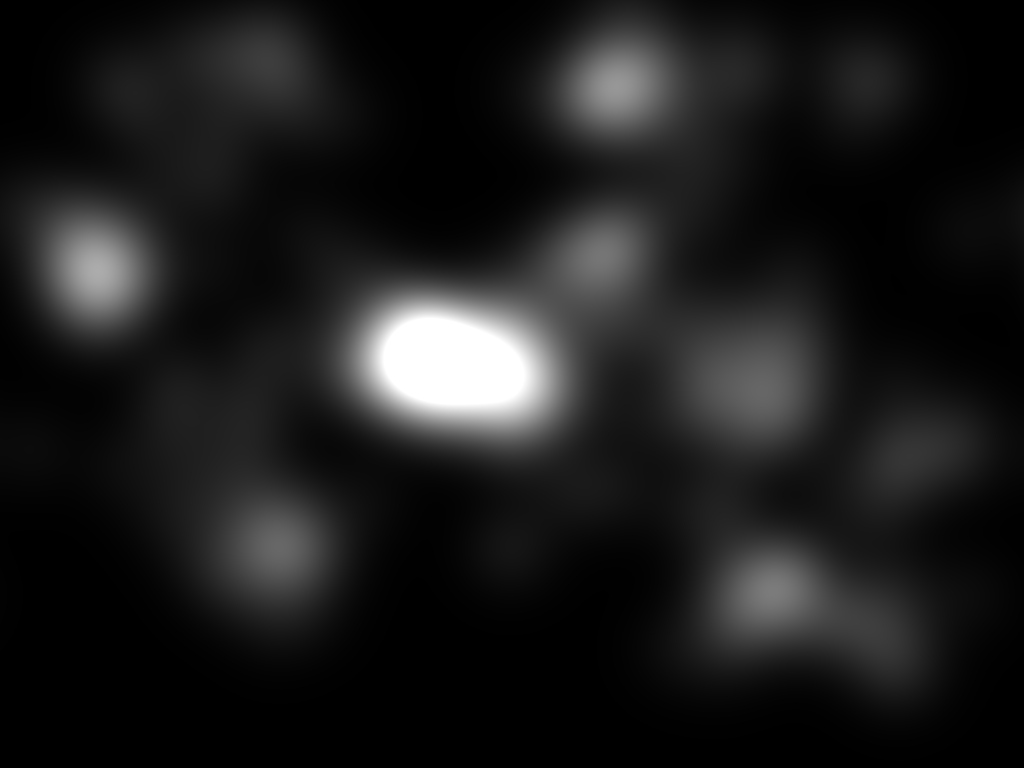

Supplement: S5 File — (ZIP) [file pone.0193149.s005.zip › S5/S5_4year.png]

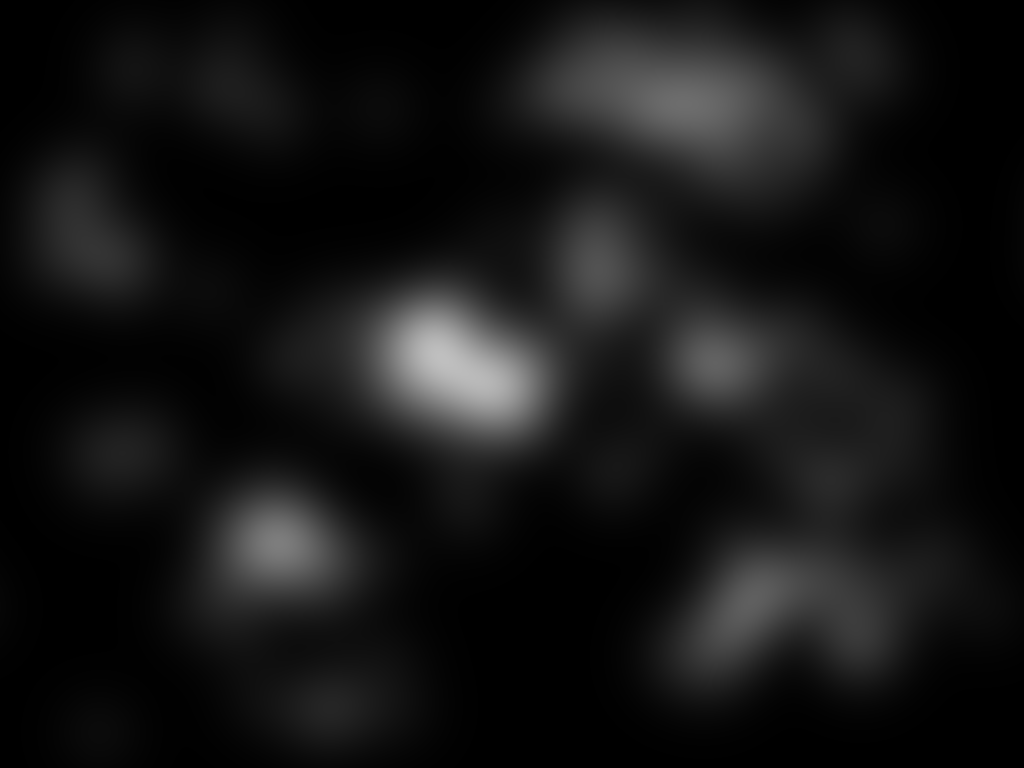

Supplement: S5 File — (ZIP) [file pone.0193149.s005.zip › S5/S5_6year.png]

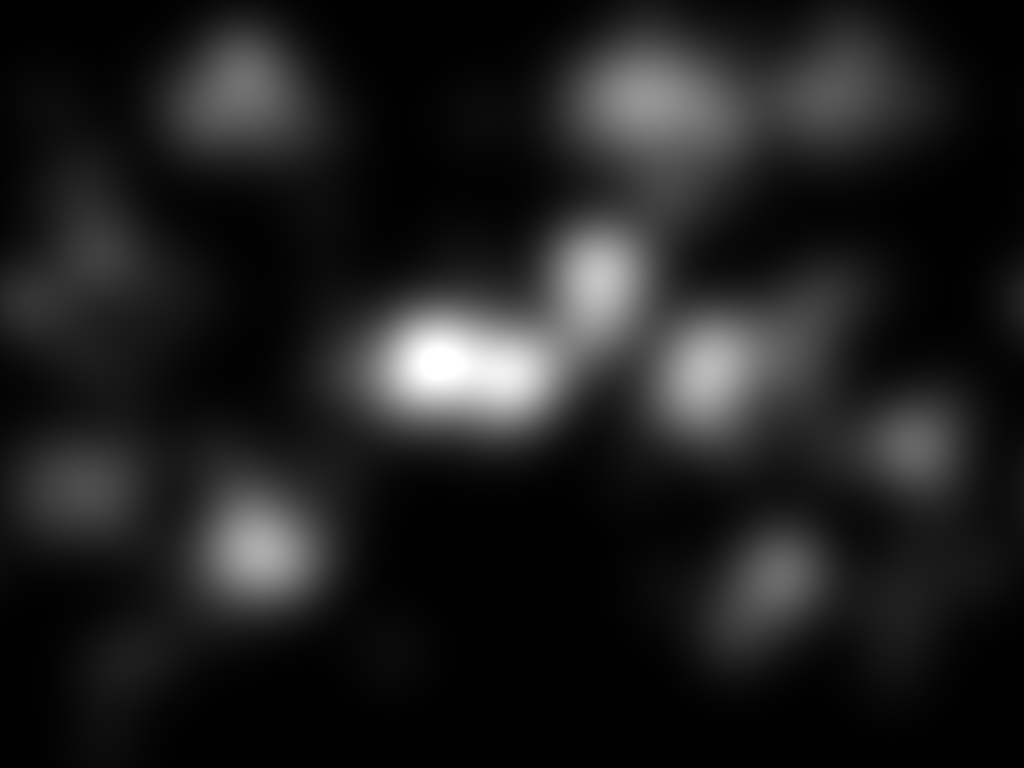

Supplement: S5 File — (ZIP) [file pone.0193149.s005.zip › S5/S5_8year.png]

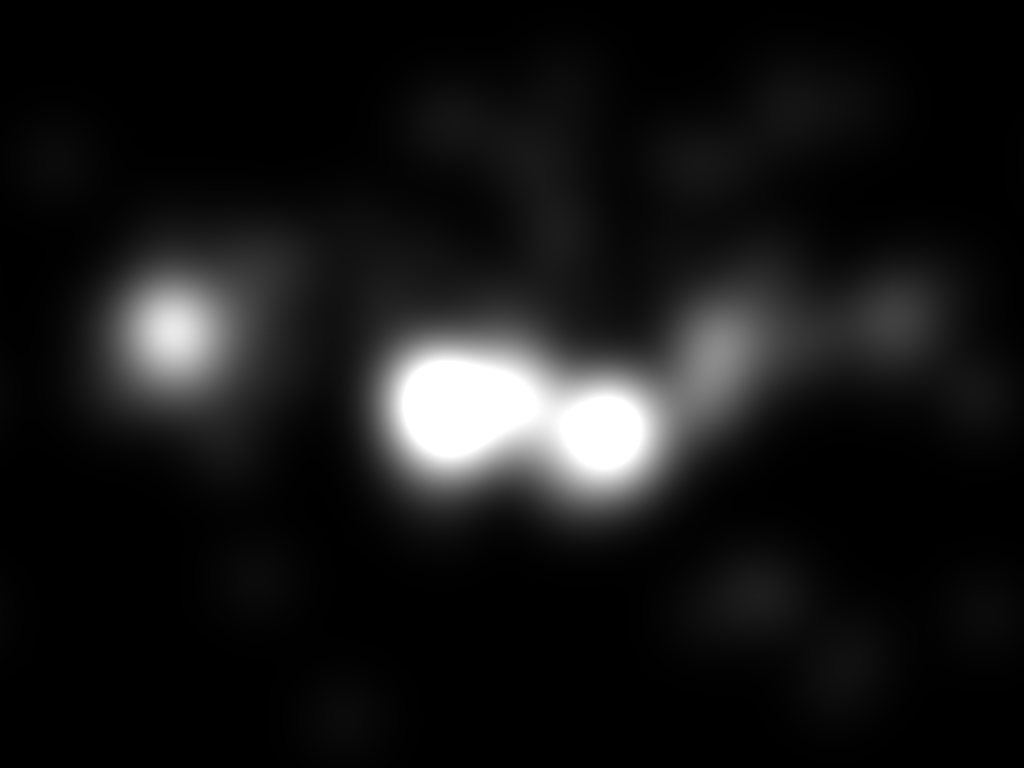

Supplement: S6 File — (ZIP) [file pone.0193149.s006.zip › S6/S6_4year.png]

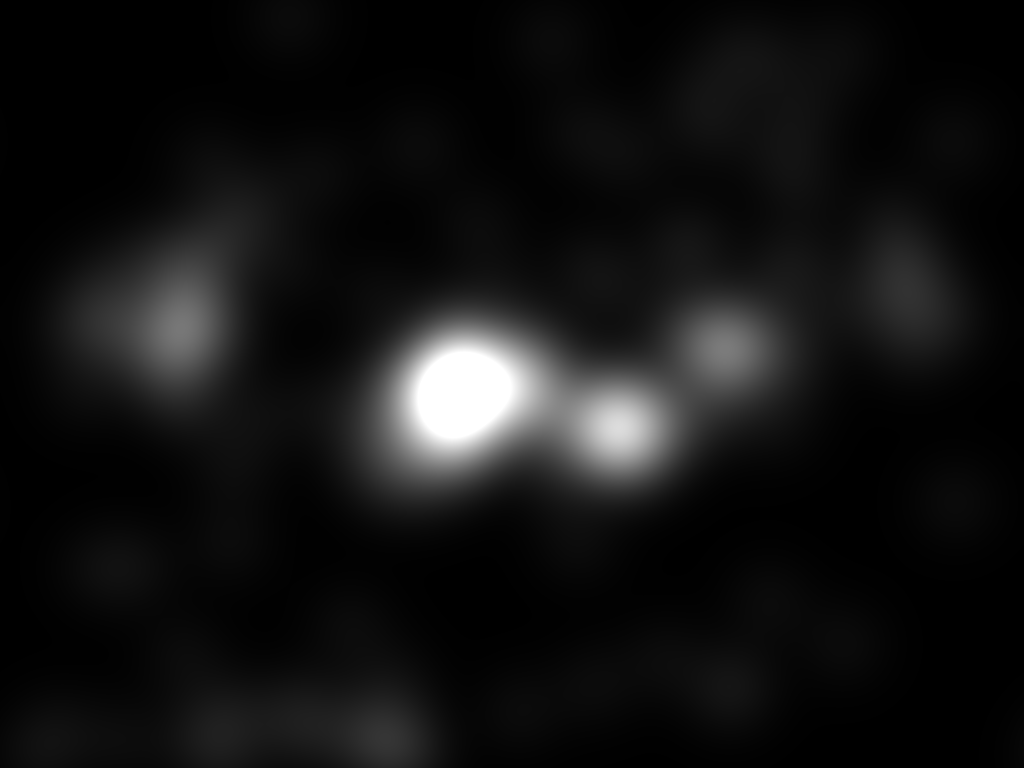

Supplement: S6 File — (ZIP) [file pone.0193149.s006.zip › S6/S6_6year.png]

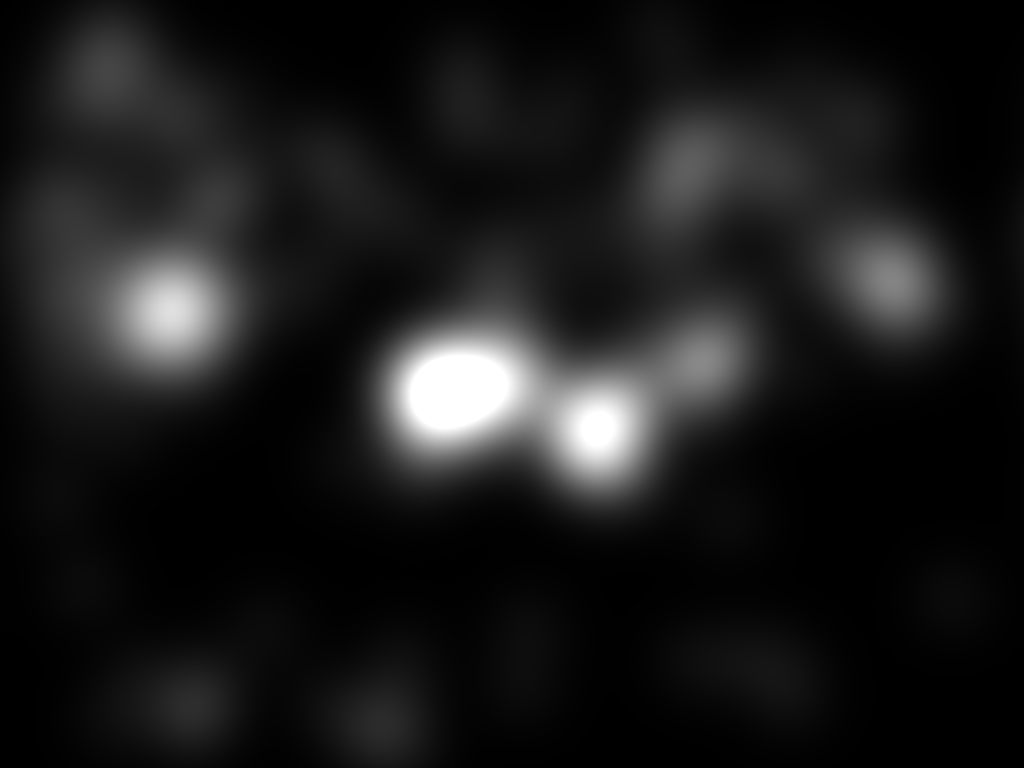

Supplement: S6 File — (ZIP) [file pone.0193149.s006.zip › S6/s6_8year.png]

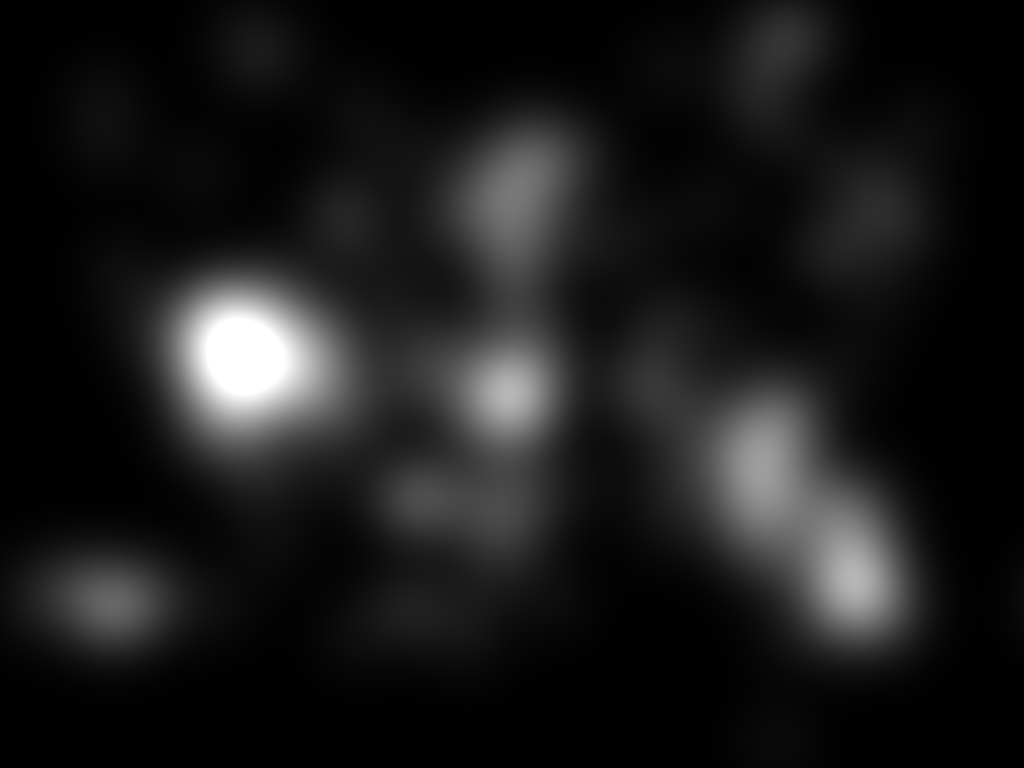

Supplement: S7 File — (ZIP) [file pone.0193149.s007.zip › S7/s7_4year.png]

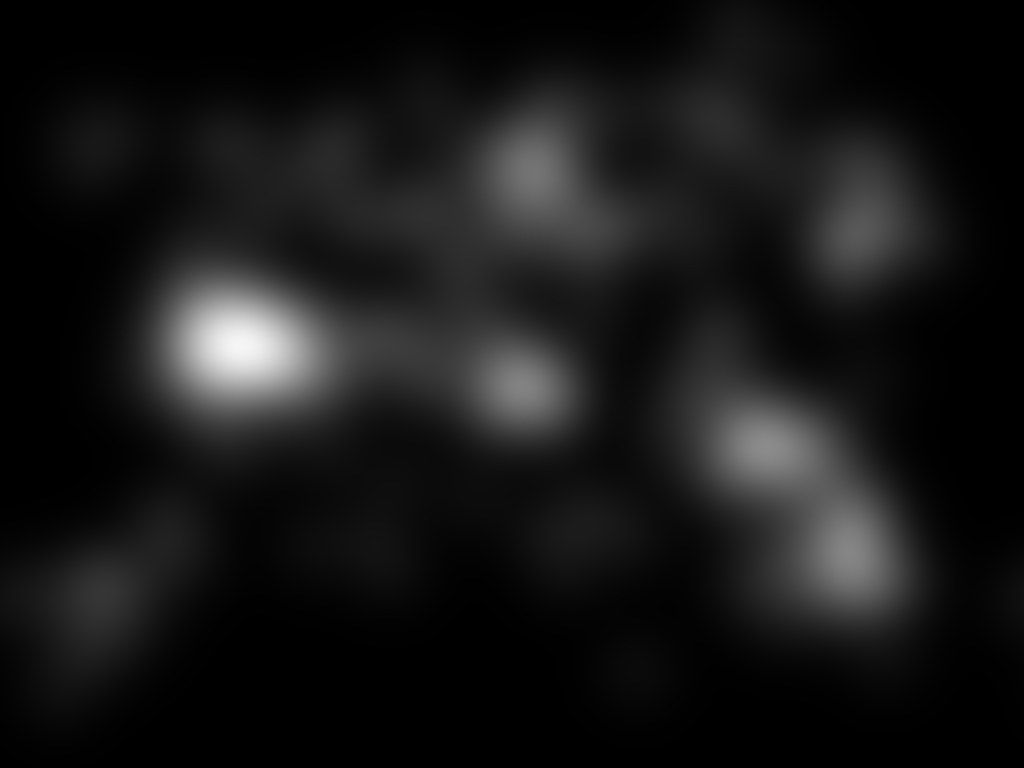

Supplement: S7 File — (ZIP) [file pone.0193149.s007.zip › S7/S7_6year.png]

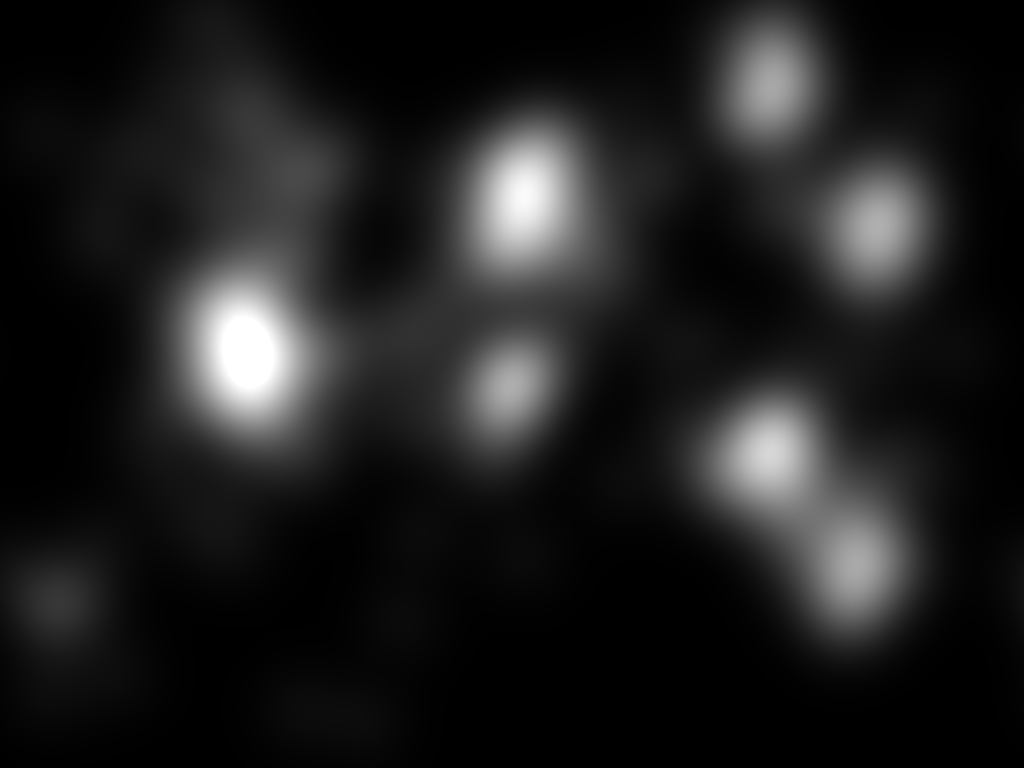

Supplement: S7 File — (ZIP) [file pone.0193149.s007.zip › S7/s7_8year.png]

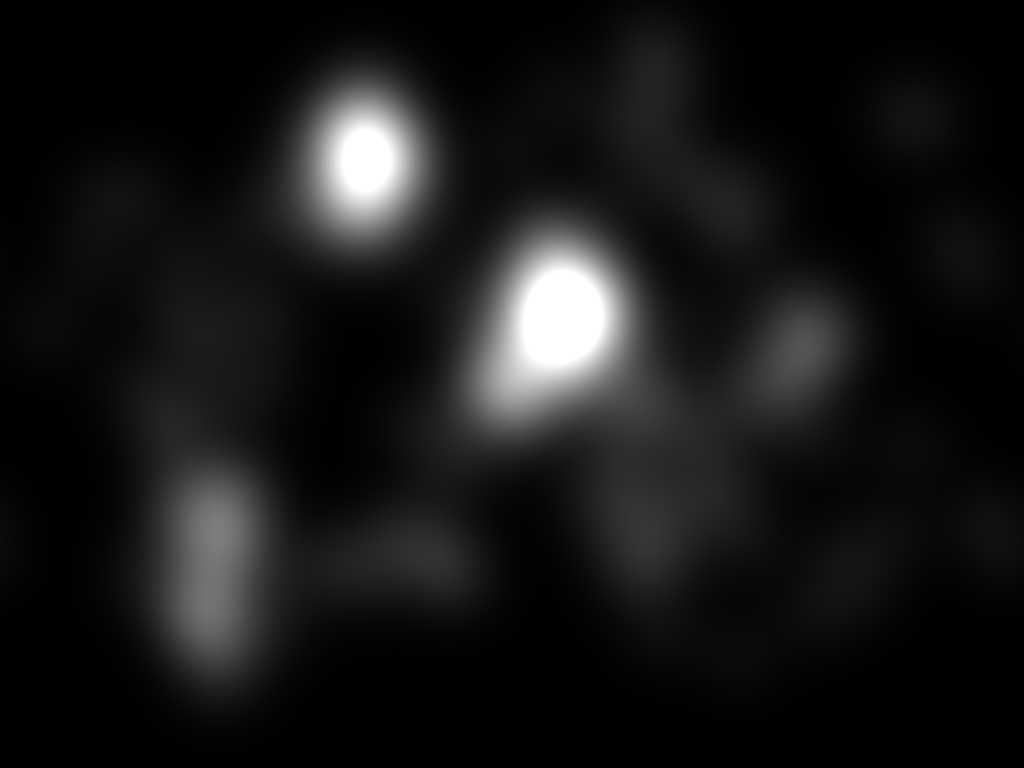

Supplement: S8 File — (ZIP) [file pone.0193149.s008.zip › S8/S8_4year.png]

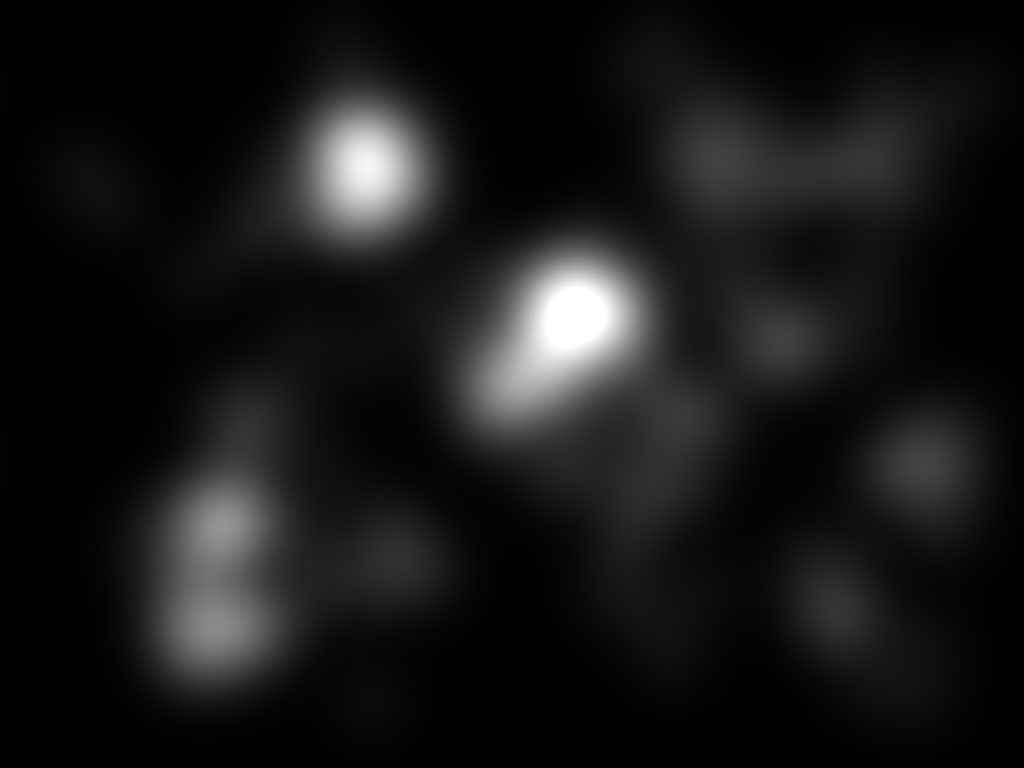

Supplement: S8 File — (ZIP) [file pone.0193149.s008.zip › S8/s8_6year.png]

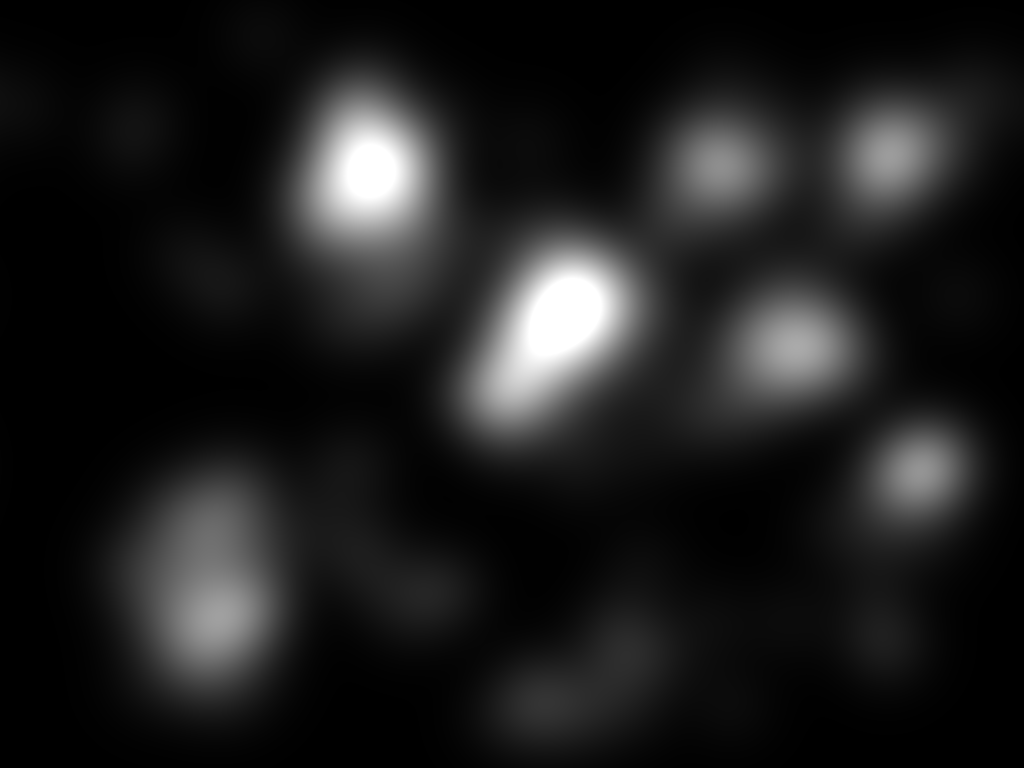

Supplement: S8 File — (ZIP) [file pone.0193149.s008.zip › S8/s8_8year.png]

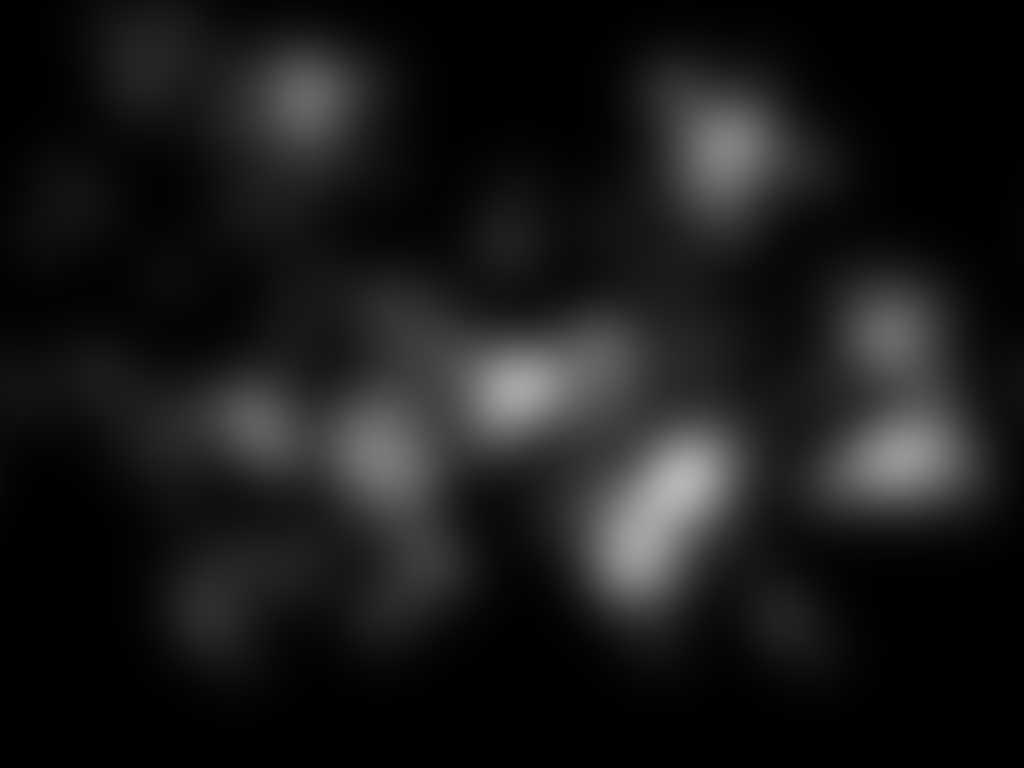

Supplement: S9 File — (ZIP) [file pone.0193149.s009.zip › S9/s9_4year.png]

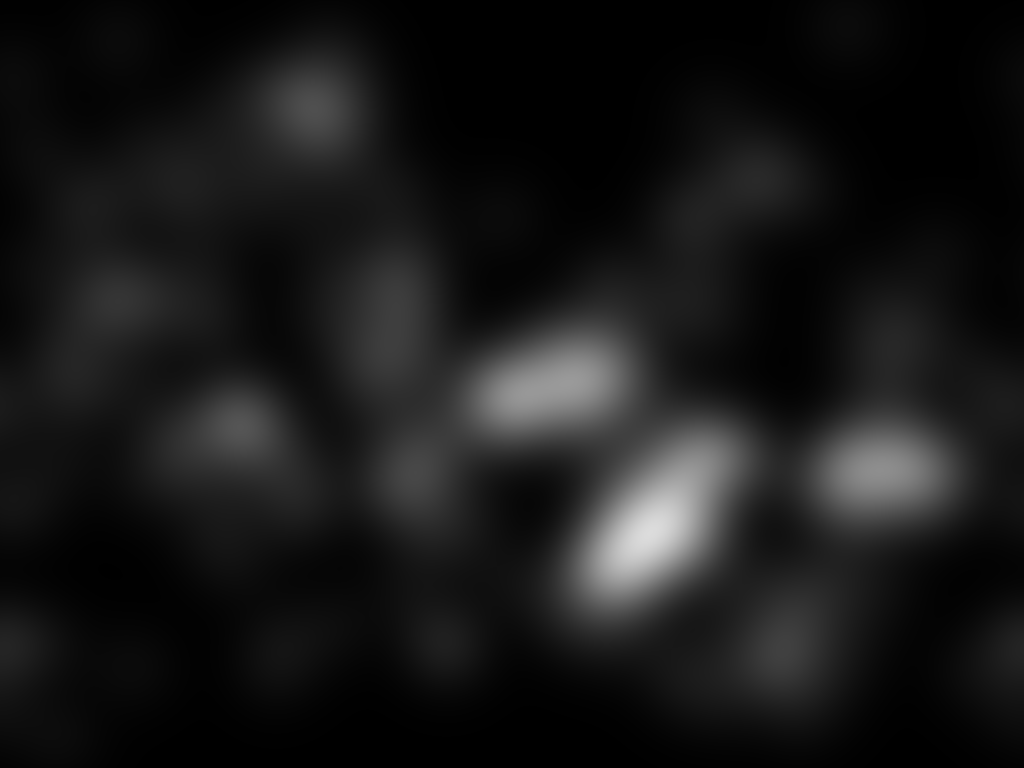

Supplement: S9 File — (ZIP) [file pone.0193149.s009.zip › S9/s9_6year.png]

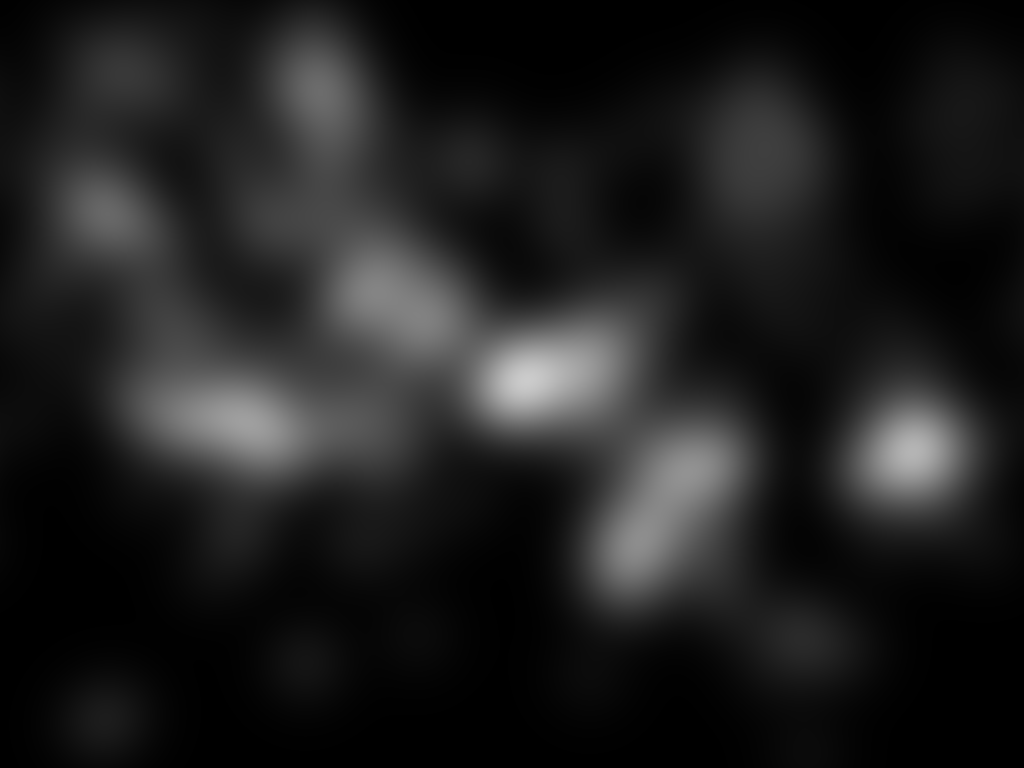

Supplement: S9 File — (ZIP) [file pone.0193149.s009.zip › S9/S9_8year.png]

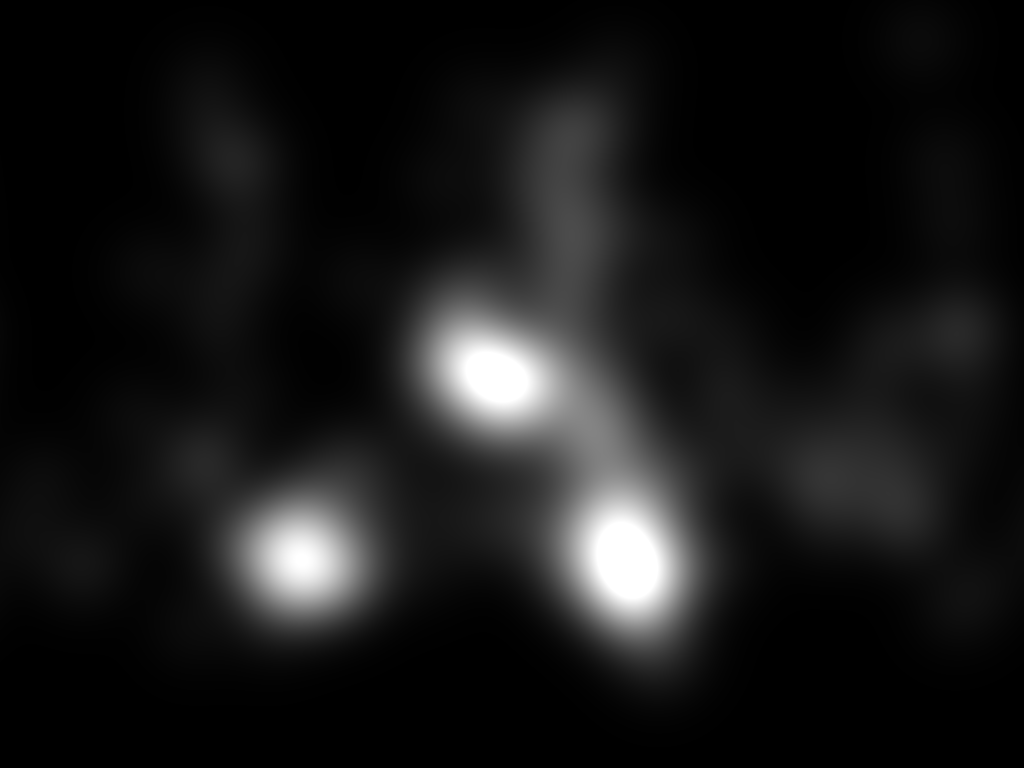

Supplement: S10 File — (ZIP) [file pone.0193149.s010.zip › S10/S10_4year.png]

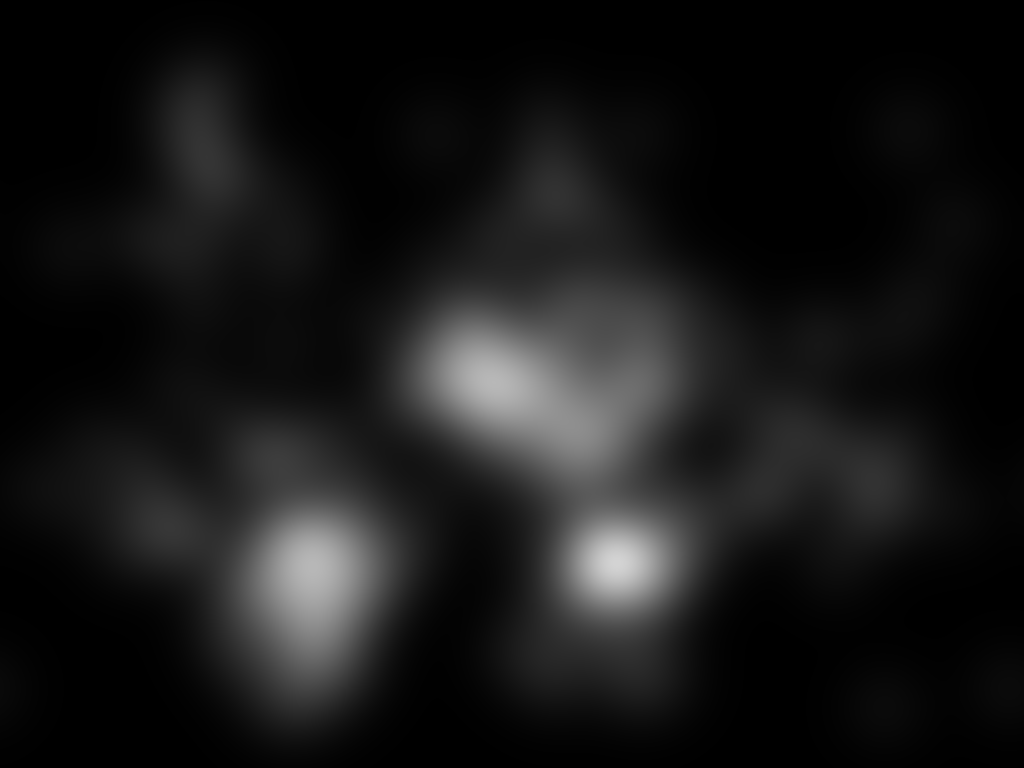

Supplement: S10 File — (ZIP) [file pone.0193149.s010.zip › S10/S10_6year.png]

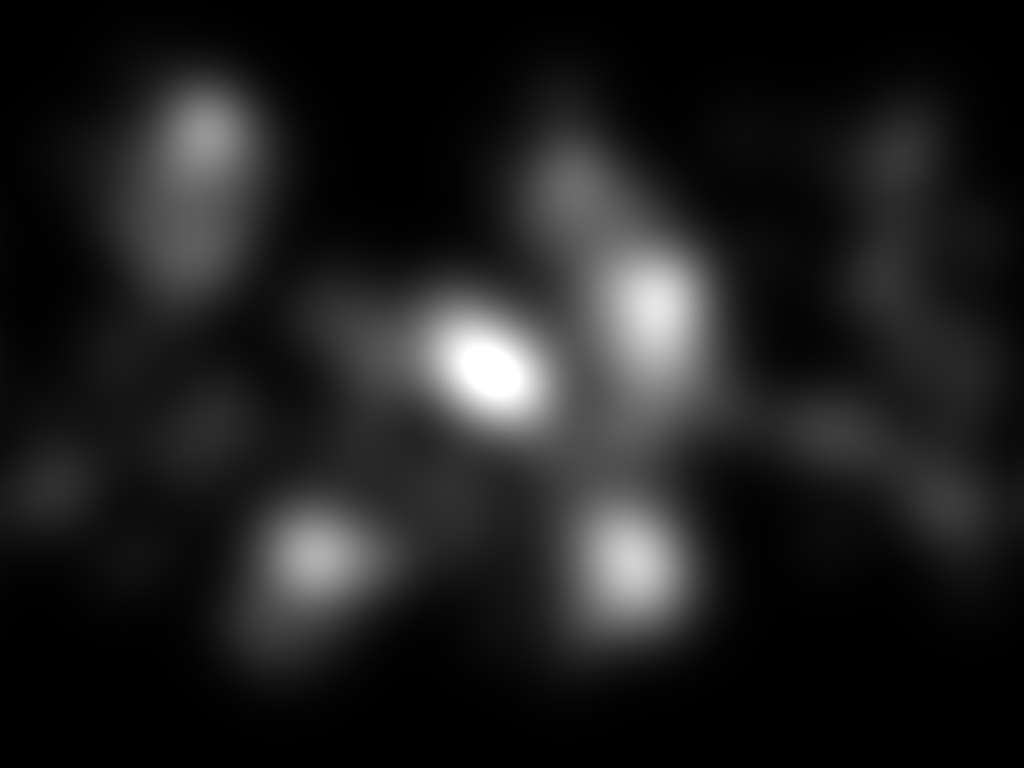

Supplement: S10 File — (ZIP) [file pone.0193149.s010.zip › S10/S10_8year.png]
